# TopoFormer: Multiscale Topology-enabled Structure-to-Sequence Transformer for Protein-Ligand Interaction Predictions

**DOI:** 10.21203/rs.3.rs-3640878/v1

**Published:** 2024-02-09

**Authors:** Dong Chen, Jian Liu, Guo-Wei Wei

**Affiliations:** 1 Department of Mathematics, Michigan State University, MI, 48824, USA; 2 Mathematical Science Research Center, Chongqing University of Technology, Chongqing 400054, China; 3 Department of Electrical and Computer Engineering, Michigan State University, MI 48824, USA; 4 Department of Biochemistry and Molecular Biology, Michigan State University, MI 48824, USA

**Keywords:** Drug design, Topological sequences, Topological Transformer, Multiscale Topology, Hyperdigraph Laplacian

## Abstract

Pre-trained deep Transformers have had tremendous success in a wide variety of disciplines. However, in computational biology, essentially all Transformers are built upon the biological sequences, which ignores vital stereochemical information and may result in crucial errors in downstream predictions. On the other hand, three-dimensional (3D) molecular structures are incompatible with the sequential architecture of Transformer and natural language processing (NLP) models in general. This work addresses this foundational challenge by a topological Transformer (TopoFormer). TopoFormer is built by integrating NLP and a multiscale topology techniques, the persistent topological hyperdigraph Laplacian (PTHL), which systematically converts intricate 3D protein-ligand complexes at various spatial scales into a NLP-admissible sequence of topological invariants and homotopic shapes. Element-specific PTHLs are further developed to embed crucial physical, chemical, and biological interactions into topological sequences. TopoFormer surges ahead of conventional algorithms and recent deep learning variants and gives rise to exemplary scoring accuracy and superior performance in ranking, docking, and screening tasks in a number of benchmark datasets. The proposed topological sequences can be extracted from all kinds of structural data in data science to facilitate various NLP models, heralding a new era in AI-driven discovery.

## 1 Introduction

The importance of discovery in modern healthcare cannot be overemphasized, as it profoundly impacts our daily lives. However, traditional methods of drug development are notably labor-intensive, consuming over a decade and costing billions of dollars for a single prescription medicine to reach the market [1]. Historically, this domain has been anchored by traditional methods such as molecular docking [2, 3, 4, 5], free energy perturbation [6], and empirically based modeling [7]. While these techniques have provided insights into drug discovery, they come with their share of limitations. Their predictive abilities also often waver in accuracy and reliability, and the computational intensity of these methods further renders them suboptimal for large-scale or swift screening endeavors. Additionally, they may overlook non-traditional binding sites or novel interaction kinetics, leading to missed therapeutic opportunities or misjudged drug efficacy.

In the evolving landscape of drug design, the deep learning models are becoming an attractive options [8, 9, 10], they have shown great capacity to predict protein structures. It was celebrated for their unmatched capability to unravel intricate patterns and deliver superior predictive outcomes. [11] This shift towards deep learning, built on the successes of chemoinformatics and bioinformatics [12], embodies the modern era’s tilt towards data-driven methodologies. However, challenges like the necessity for frequent retraining and an overwhelming reliance on labeled data have been persistent roadblocks.

The groundbreaking Transformer framework and models like ChatGPT, which owe their triumphs to large-scale pre-training and the adept use of unlabeled data, point towards the untapped potential of self-supervised learning [13, 14, 15]. These models offer a glimpse of powerful solutions, especially when traditional labeled data is a limiting factor. While the success of the Transformer framework in the realm of natural language processing is undeniable, its direct application to the domain of drug discovery, especially for the protein-ligand complex modeling, raises pertinent questions because of its neglecting important stereochemical relations. One pivotal quandary is tailoring a model, intrinsically designed for serialized language translations, to suit the study of protein-ligand complexes, which inherently defy serialized representation.

**Figure 1:**
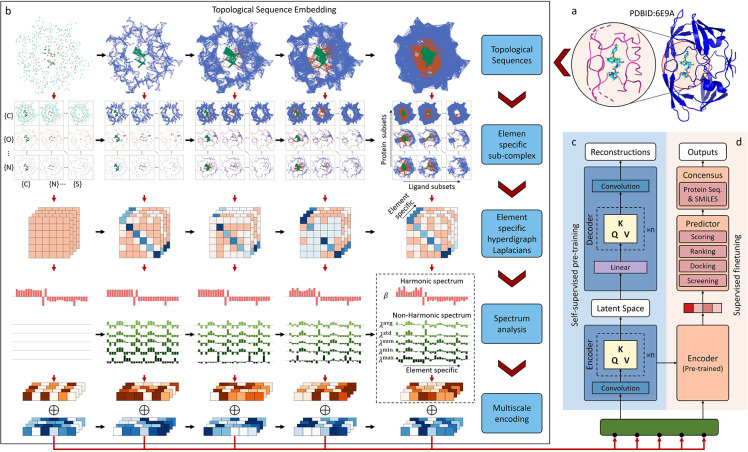
Schematic illustration of the overall TopoFormer model. **a**, A 3D protein-ligand complex (PDBID: 6E9A) and its interactive domain. **b**, The topological sequence embedding of a 3D protein-ligand complex. Initially, the complex is split into a topological sequence, known as a chain complex in algebraic topology. Then, element-specific sub-complexes are created to encode physical interactions a variety of scales controlled by a filtration parameter. Subsequently, element-specific persistent topological hyperdigraph Laplacians (PTHLs) are utilized to extract the topological invariant and capture the shape and stereochemistry of the subcomplexes. For these subcomplexes, their topological invariant changes over scales are retained in the harmonic spectrum of the hyperdigraph Laplacians, while their homotopic shape evolution over scales are manifested in the non-harmonic spectrum. Finally, the multiscale topological invariant changes and homotopic shape (stereochemical) evolution are resembled into a topological sequence as the input the Transformer. **c**, Self-supervised learning is applied to unlabeled topological sequences for both Transformer Encoders and Transformer Decoders. The outputs from the reconstructed topological sequences are used to calculate the reconstruction loss. **d**, At the supervised fine-tuning stage, task-specific protein-ligand complex data are fed into the pretrained encoder, which is equipped with specific predictor heads, such as the Scoring head, Ranking head, Docking head, and Screening head. Subsequently, except for the docking task, the remaining predictions are consolidated with sequence-based predictions to produce the final result.

In response to the existing challenges, we leverage advanced mathematical models from algebraic topology, differential geometry, and combinatorial graph theory. These models, previously applied to represent biomolecular systems, have achieved significant successes [16, 17, 18, 19]. Drawing upon unique insights from advanced mathematics, we unveil our topological transformer model: TopoFormer. TopoFormer is built upon persistent topological hyperdigraph Laplacian (PTHL) [20], a tarnsformative algebraic topological model. While intrinsically mirroring foundational topological invariants akin to traditional persistent homology [21], this multiscale technique introduced the novel topological hyperdigraph to capture intrinsic physical, chemical, and biological interactions in protein-ligand binding, and uniquely delivers a non-harmonic spectrum, shedding light on the three-dimensional (3D) shape intricacies of protein-ligand complexes. In a nutshell, PTHL utilizes its multiscale topology and multiscale spectrum to convert intricate 3D protein-ligand complexes into 1D topological sequences that are ideally suitable for the sequential architecture of Transformers (Figure 1). This innovative fusion not only melds topological insights with cutting-edge machine learning but also heralds a paradigm shift in our grasp of protein-ligand relationships. Capitalizing on its deep-rooted topological framework, TopoFormer redefines performance benchmarks in drug research tasks like scoring, ranking, docking, and screening. Its nuanced design ensures that unconventional interactions are not overlooked but are instead spotlighted. As shown in the results, TopoFormer consistently outshines its peers, achieving state-of-the-art outcomes across diverse benchmark datasets in drug discovery.

## 2 Results

In this section, an overview of the proposed topological transformer (TopoFormer) model is provided, followed by a comprehensive evaluation of its performance across crucial tasks, including scoring, ranking, docking, and screening. The analysis contextualizes TopoFormer’s capabilities within the framework of existing methodologies, thus revealing both the strengths and advantages of this novel model when compared to established techniques.

### 2.1 Overveiw of TopoFormer for protein-ligand binding analysis

Transformer [13] architecture offered a groundbreaking technique that leverages attention mechanisms to understand sequential data in various domains [14, 22, 23]. Drawing inspiration from the Transformer’s design and capabilities, we have conceived a topological transformer model named TopoFormer, as shown in Figure 1. TopoFormer integrates our new persistent topological hyperdigraph Laplacian (PTHL) [20] and transformer for the first time. Unlike other Transformers that are based on protein and ligand sequence information, TopoFormer takes 3D protein-ligand complexes as inputs. This is made possible through the unique transformation of intricate 3D protein-ligand complexes into sequences of topological invariants and homotopic shape and stereochemical evolution by PTHL. The PTHL technique sequentially embeds the topological invariants, the homotopic shape, and the physical, chemical, and biological interactions of 3D protein-ligand complexes at various scales into a topological sequence admissible to the transformer architecture. Pretraining on a diverse set of protein-ligand complexes empowers the model to grasp the broad characteristics and nuances of molecular interactions, including various sterochemical effects that cannot be captured by traditional molecular sequences. Subsequent fine-tuning on specific datasets ensures that the output embeddings for each complex not only capture the intrinsically intricate interactions within the complex but also represents the traits of the complex in contact with the whole dataset, which facilitates the downstream deep learning.

To define a specific domain for our analysis, we firstly pinpoint all heavy ligand atoms and the protein atoms within a predetermined distance, as shown in Figure 1a. And two versions of the model are available: one with a generous 20 Å cutoff and another with a 12 Å cutoff (suited for a more focused analysis). Next, in order to convert 3D molecular structures to admissible format, TopoFormer applies its unique topological sequence embedding module, as shown in Figure 1b. By employing a multiscale analysis, also known as a filtration process in algebraic topology, the 3D structures are transformed into topological sequences using our newly developed persistent topological hyperdigraph Laplacians (PTHLs). We further embed various physical, chemical, and biological interactions element-specific PTHLs. The outcome is a sequence of embedding vectors, enabled through the multiscale analysis of PTHLs. A more detail description of the topological sequence embedding module can be found in the methods section 4.2.

To take the advantages of a vast variety of unlabeled protein-ligand complexes, TopoFormer utilizes a self-supervised pretraining phase as depicted in Figure 1c. At its core lies the Transformer encoder-decoder architecture, wherein the decoder diligently aims to reconstruct the topological sequence embedding from its encoded version. The precision of this phase is quantified by measuring the disparity between the output and input embeddings. Absent of labeled annotations, this step equips the model with an innate ability to decipher the intricate dynamics of protein-ligand interactions. Subsequent to pretraining, as illustrated in Figure 1d, the model acquaints itself with labeled protein-ligand complexes, transitioning to a supervised fine-tuning stage. Leveraging the pretrained encoder, the foremost embedded vector evolves into a pivotal latent feature, guiding a plethora of downstream tasks. Among TopoFormer’s distinguishing attributes is its proficiency in executing multiple tasks, encompassing scoring, ranking, docking, and screening. Each task is equipped with its specialized head within the predictor module. To enhance precision, several topological transformer deep learning models (TF-DL) are initiated, each with a unique random seed, to mitigate initialization-related inaccuracies. Additionally, to temper the inherent biases of relying solely on one modeling approach, sequence-based models are also incorporated. Consequently, the conclusive output of TopoFormer is derived as an amalgamation of these varied predictions. The consensus methodology for each task will be elaborated upon in the subsequent task-specific results. In essence, TopoFormer is a holistic model tailored for a myriad of tasks in protein-ligand interaction analysis, bringing together topological insights and deep learning.

### 2.2 Evaluating TopoFormer on scoring tasks

The prediction of protein-ligand binding affinity plays a pivotal role in drug design and discovery. To assess the scoring capability of our models, we have evaluated them using the three most widely recognized protein-ligand datasets from the PDBbind database: CASF-2007, CASF-2013, and CASF-2016 [24, 25, 26]. The Pearson correlation coefficient (PCC) and the root mean squared error (RMSE) are used to measure the performance of the scoring function. For the kcal/mol unit conversion, we multiply the predicted values by 1.3633 in the predictions. In this task, we consider two TopoFormer models: a large model (TopoFormer) with an input topological sequence of length 100, employing filtration parameters at 0.1 intervals spanning from 0 Å to 10 Å. The topological analysis encompasses a domain extending up to 20 angstroms, centered at the ligand. Additionally, we employ a smaller model (TopoFormer_*s*_) with an input topological sequence of length 50, using filtration parameters ranging from 2 angstroms to 12 angstroms, and with filtration parameter increments of 0.2 angstroms for constructing the corresponding simplex complex. The topological analysis covers a domain up to 12 angstroms, centered at the ligand.

**Figure 2:**
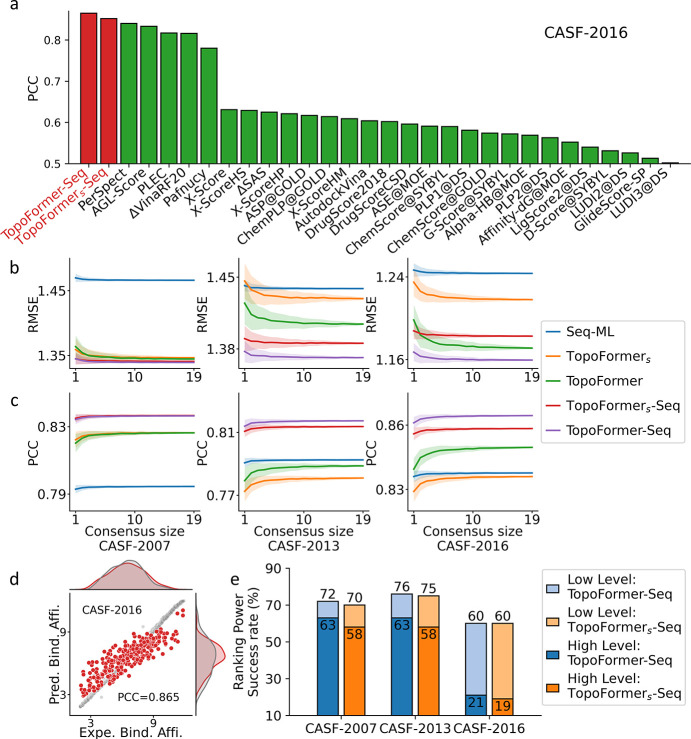
Performance of TopoFormer on scoring and ranking tasks. **a** Comparison of Pearson correlation coefficients (PCCs) of various models for protein-ligand complex binding affinity scoring on the CASF-2016 benchmark. The results from other methods are in the green color, taking from Refs [25, 24, 26, 16, 27, 19, 28, 29, 30]. **b** Comparison of the RMSEs of predictions for the CASF-2007, CASF-2013, and CASF-2016 datasets from the Seq-ML model, TopoFormer model, TopoFormer_*s*_ model, TopoFormer_*s*_-Seq, and TopoFormer-Seq. The horizontal axis is the number of models in the consensus (consensus size). The solid line represents the median RMSE, while the shaded background provides the error bar for these 400 RMSE values. **c** Comparison of the PCCs of predictions for the CASF-2007, CASF-2013, and CASF-2016 datasets from the Seq-ML model, TopoFormer model, TopoFormer_*s*_ model, TopoFormer_*s*_-Seq, and TopoFormer-Seq. The horizontal axis is the consensus size. The solid line represents the averages, while the shaded background provides the error bar for 400 PCCs at each consensus size. **d** The correlation between predicted protein-ligand binding affinities (TopoFormer PCC=0.865) and experimental results for the CASF-2016 benchmarks. Grey dots represent the training data, while red dots denote the test data. **e**, Comparison of the ranking power assessed using both high-level success measurements (depicted in dark shades) and low-level success measurements (shown in lighter shades) across three benchmarks. Results from TopoFormer-Seq are represented in blue, while those from TopoFormer_*s*_-Seq are illustrated in orange.

To ensure robustness, 20 topological transformers are trained for each dataset with distinct random seeds to address initialization-related errors. Here, the predictions only from small topological transformers are denoted as TopoFormer_*s*_. In addition, to attenuate systematic discrepancies inherent in a singular model approach, we deploy sequence-based models. Specifically, we harness embedded protein features from the ESM model [31] and the SMILES features from the Transformer-CPZ model [22]. And 20 gradient boosting regressor tree (GBRT) models are subsequently trained one these sequence-based features. The aggregated predictions from these models, denoted as Seq-ML, render a more holistic prediction. Thus, the final verdict results from a balanced average of TopoFormer and Seq-ML predictions, denoted as TopoFormer-Seq and TopoFormer_*s*_-Seq for small TopoFormer model. Figure 2b and Figure 2c show the effect of consensus size (i.e., the number of randomly selected models) on performance. We performed 400 repetitions for each consensus size, taking the average result (solid line) and showing error variation (lighter-colored regions). It can be seen that the increasing in the consensus size improved performance metrics (higher PCC, lower RMSE) and stability (reduced error fluctuation). Ultimately, the consensus size is fixed as 10 for the subsequent comparisons. It can also be noticed that the TopoFormer-Seq performs best on almost all datasets, closely followed by the TopoFormer_*s*_-Seq model.

To gain a comprehensive understanding of our models’ performance, we benchmark our PCC results against representative results from the literature, as visualized in Figure 2a and Figure 7a–b. Remarkably, our TopoFormer-based models consistently achieved the highest PCC scores across all three benchmark datasets. The RMSE of our model is also the lowest in all three benchmark datasets when compared to methods with accessible RMSE (1). In this work, the TopoFormer-based model’s performance is quantified by calculating averages from 400 repetitions, and the results are tabulated in Table 1. Across all three datasets, Transformer-Seq achieves an average PCC of approximately 0.84. For a detailed comparison of various models trained on the same dataset, please refer to Table S1. Notably, in the case of the PDBbind v2016 dataset, [26] which has five more components (290) in its test set compared to the CASF-2016 core set (285), our TopoFormer-Seq model also demonstrated state-of-the-art performance with a PCC of 0.866 and a low RMSE of 1.561 kcal/mol. The detailed information of these three benchmarks can be found in Table S 2. Figure 2d, and Figure 7c–d visualized the comparisons of predicted protein-ligand binding affinities and experimental results for the test set of CASF-2007, CASF-2013, and CASF-2016 benchmarks.

Recently, several deep learning models have been reported for the prediction of protein-ligand binding affinity. Notable examples include the graphDelta model[32], ECIF model[33], OnionNet-2 model[34], DeepAtom model[35], and others[36, 37, 38]. These new models typically leverage on large training datasets that incorporate additional data from the general sets of the PDBbind database and thus are not comparable with other models that were trained on different training datasets. The details regarding the composition of training sets, testing sets, and their corresponding performance, are tabulated in Table S 5. In this study, the proposed TopoFormer model was trained strictly on the refine dataset following the standard procedure [24, 25, 26], representing a smaller subset compared to the general set. The results obtained with TopoFormer, trained on the refine set (which is notably smaller), outperform the majority of these models. Furthermore, for the latest PDBbind v2020 [39], we consider a total of 18,904 protein-ligand complexes for training, which has no overlap with the core sets of CASF-2007, CASF-2013 and CASF-2016. Our model achieved a commendable final PCC of 0.853 and an RMSE of 1.295 (equivalent to 1.769 kcal/mol) on the core set of CASF-2007. For the CASF-2013 core set, the PCC of 0.832 and an RMSE of 1.301 (equivalent to 1.777 kcal/mol) are obtained. Similarly, on the CASF-2016 core set, we obtained a PCC of 0.881 with an RMSE of 1.095 (equivalent to 1.496 kcal/mol). For the PDBbind v2016 core set, we achieved a PCC of 0.883 with an RMSE of 1.086 (equivalent to 1.483 kcal/mol). Here, all the results are the average of 400 repeated experiments. These results underscore the robustness and predictive power of the TopoFormer model in the realm of protein-ligand binding affinity predictions.

### 2.3 Evaluating TopoFormer on ranking tasks

The efficacy of a scoring function is critically assessed by its aptitude to accurately rank the binding affinities of protein-ligand complexes within distinct clusters. The benchmarks CASF-2007 and CASF-2013 comprise 65 clusters, with each cluster containing three complexes formed by an identical protein partnered with varied ligands [25, 24]. On the other hand, the CASF-2016 benchmark encompasses 57 clusters, each having five distinct complexes [26]. In this work, two evaluative approaches are employed: the high-level and the low-level success measurements. In the high-level success metric, the objective is to perfectly rank the binding affinities of the complexes within each cluster. Conversely, the low-level success criterion requires the scoring function to merely identify the complex with the pinnacle binding affinity. The assessment of ranking efficacy termed “ranking power” is gauged by the proportion of correctly identified affinities across a specified benchmark. The mathematical formulations of the high-level and low-level success measurements can be found in the Supplementary Materials Section A.1.

Figure 2e illustrates the ranking power of TopoFormer-based models. For the CASF-2007, the TopoFormer-Seq model achieved outstanding success rates, with 72% for low-level measurement and 63% for high-level measurement. In comparison, the TopoFormer_*s*_-Seq model achieved success rates of 70% for low-level and 58% for high-level measurement. Both models outperformed previous approaches, as demonstrated in high-level measurement Figure S8 and low-level measurement Figure S9. Similarly, for the CASF-2013, the TopoFormer-Seq model achieved remarkable success rates of 76% for low-level and 63% for high-level measurement, surpassing the performance of earlier models. The challenges intensified in CASF-2016, comprising 57 clusters, each containing five distinct complexes [26], making ranking tasks notably more demanding. In this context, the TopoFormer-Seq model achieved a success rate of 60% for low-level measurement and 21% for high-level measurement. The best-performing models for low-level (68%) and high-level (29%) success were ΔVinaRF20 [30].

### 2.4 Evaluating TopoFormer on docking tasks

Molecular docking stands as a formidable computational tool, essential in the fields of drug discovery, structural biology, and the elucidation of molecular intricacies underlying biological processes. The pivotal role of a robust scoring function becomes evident when selecting the most promising binding poses and predicting binding affinities. In the present study, we harnessed the capabilities of TopoFormer_*s*_ (Due to computational resource constraints, we only employed TopoFormer_*s*_ for both docking and screening tasks.) to assess its docking proficiency, particularly its ability to distinguish native binding poses from those generated by established docking software packages. Our evaluation centered on benchmark datasets CASF-2007 and CASF-2013 [25, 24]. Each dataset comprises a total of 195 test ligands, with each ligand accompanied by 100 poses generated by various docking programs. A pose was considered native if its root mean square deviation (RMSD) with respect to the true binding pose was less than the 2 Å threshold. Successful prediction occurred when the pose with the highest predicted binding energy matched a native pose. Following this comprehensive evaluation encompassing all 195 test ligands, an overall success rate was computed for the employed scoring function. Additional information detailing the assessment of docking success rates is available in the Supplementary Information Section S A.1.

**Figure 3:**
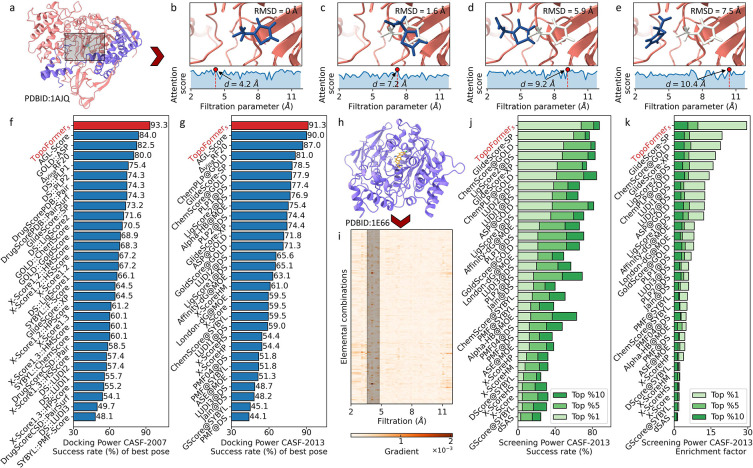
Performance of TopoFormer on docking and screening tasks. **a**, Visualization of the protein-ligand complex PDBID: 1AJQ. The highlighted rectangle shows the protein’s pocket area. **b**-**e**, Four distinct ligand poses within the protein 1AJQ. The molecule in light gray represents the true pose, while the blue molecules depict alternative poses with RMSD values of 0 Å, 1.6 Å, 5.8 Å, and 7.5 Å, respectively. The light blue curve represents the attention score generated by TopoFormer, varying with the filtration parameter (i.e., the scale) of the topological embedding. The highest attention scores are observed at scales of d=4.2Å,d=7.2Å,d=9.2Å, and d=10.4Å for poses from **b** to **e**. **f**-**g**, Comparison of docking success rates between TopoFormer_*s*_ and traditional docking tools on the CASF-2007 core set (**f**) and the CASF-2013 core set (**g**). **h**, Visualization of the protein-ligand complex PDBID: 1E66. **i**, The saliency map of the topological embedding for complex 1E66. The colorbar represents the gradient weights of each feature relative to the prediction. **j**, Comparison of screening success rates for the top 1%, top 5%, and top 10% selected ligands between TopoFormer_*s*_ and docking tools on the CASF-2013 core set. **k**, Comparison of average enhancement factors for the top 1%, top 5%, and top 10% selected ligands between TopoFormer_*s*_ and docking tools on the CASF-2013 core set.

In the field of molecular docking, several noteworthy approaches have made significant strides, each contributing uniquely to our understanding of protein-ligand interactions. Notable among these are DeepDock [40], which achieved a commendable success rate of 62.11%, OnionNet-SFCT [41] further enhanced performance to an impressive 76.84%, followed by DeepBSP [42] at 79.7%, and RTMScore [43] reaching a remarkable 80.7% success rate on the PDBbind core set. It is noteworthy that these methodologies were trained on diverse datasets, making direct comparisons challenging. In the pursuit of a comprehensive evaluation, we utilized the publicly available training data to train the TopoFormer_*s*_. We then conducted a rigorous comparison on the CASF-2007 and CASF-2013 datasets, fostering a fair and unbiased assessment of our methodology [27, 30, 44]. Detailed pose data and labels are provided in Section 4.1. Impressively, as depicted in Figures 3f and 3g, TopoFormer_*s*_ attained an exceptional success rate of 93.3% on the CASF-2007 core set and 91.3% on the CASF-2013 core set. TopoFormer_*s*_ outperformed other established docking tools and models, highlighting the effectiveness of our topological approach. Our methodology stands as a testament to the richness of approaches in the field, harnessing innovative techniques and a meticulously curated dataset to achieve remarkable success rates in docking tasks, while ensuring fairness in comparison. It offers a fresh perspective and a robust toolkit for the docking challenge.

In order to better understand what Topoformer_*s*_ learned from training after fine-tuning, we explored which filtration parameter, i.e., the spatial scale, had the greatest impact on protein-ligand interactions through attention scores. Figures 3b–e show the four poses of the ligand in the vicinity of the protein (PDBID: A1JQ) pocket (black boxed portion in Figure 3a). Where Figure 3b is the real pose measured by experiment, which has an RMSD of 0 Å. After training, we obtain the TopoFormer_*s*_’s attention score for all filtration parameters, i.e., the average of the attentional weights of all heads in all TopoFormer layers. This attention score indicates the magnitude of the impact of the protein-ligand interaction of each range on the final docking score. From Figure 3b, it can be noticed that the largest attention score occurs at d=4.2Å, which generally indicates that the interactions at ranges with the scale of 4.2 Å have the largest impact on the binding affinity of this pose. Similarly, Figure 3c–e show poses with RMSDs of 1.6 Å, 5.9 Å, and 7.5 Å, respectively, where the light gray compounds refer to ligand’s pose when the RMSD is zero. Their corresponding maximum attention score, on the other hand, occur at scales d=7.2Å,d=9.2Å, and d=10.4Å, respectively, which are positively correlated with RMSDs. It indicates that the more a pose deviates from the true pose position, the greater the scale at which the interactions have the greatest impact on the docking score is.

### 2.5 Evaluating TopoFormer on screening tasks

The screening task in biology is of paramount importance in identifying potential drug candidates and advancing drug development endeavors. To assess the screening capabilities of our TopoFormer method, we employ the CASF-2013 core set in this study. Given that the evaluation of screening power necessitates the identification of three true binders for each of the 65 proteins in the core set, we take the crucial step of fine-tuning the pre-trained TopoFormer_*s*_ model. For this purpose, we assemble a training dataset encompassing both ligand poses and energy labels, customizing TopoFormer_*s*_ for each protein target. Our screening task comprises two key steps. First, we generate poses for the 195 ligands through a docking procedure and predict their scores using TopoFormer_*s*_, denoted as $S_{1}$. Subsequently, we employ a sequence-based classification gradient boosting decision tree model, leveraging combined features from the Transformer-CPZ model [22] and the ESM model [31] for these 195 ligands and the respective target proteins. This yields probabilities for the given ligands, referred to as $S_{2}$. Ligands with high multiplied scores $\left(S=S_{1}*S_{2}\right.$ are identified as predicted binders. Consistent with prior research, the training set for each target protein comprises all complex structures and their associated energy labels from the PDBbind v2015 refine set, excluding the core (test) set complexes. Furthermore, for each target protein, additional poses and their corresponding labels in the training set are generated [45, 27]. Comprehensive pose data and labels for the screening task can be found in Section 4.3. Here, due to computational resource constraints, we only utilize TopoFormer_*s*_ for virtual screening. Additionally, in this work, the success rate and enrichment factor (EF) are used in the virtual screening for drug discovery. The success rate measures the proportion of true positive predictions among the top-ranked compounds. And the enrichment factor is a measure of how well a screening method enriches the dataset with active compounds (true binders) at the top of the ranked list, specifically 1%, 5%, and 10%, compared to a random selection. It provides insight into the ability of the method to prioritize active compounds over non-active ones. The detailed definitions for both success rate and enrichment factor are provided in Supplementary Information Section SA.1.

As suggested by Figures 3j and 3k, the proposed TopoFormer model outperformed the previous methods in all two metrics, e.g., success rate and enrichment (EF), compared with popular conventional methods. Concretely, for the task of identifying true binders for a certain protein, TopoFormer attains a success rate of 68% and an average enhancement factor (EF) of 29.6% on top 1%-ranked molecules. It was better than AGL-score [27] (success rate=68%, EF=25.6) and ΔVineRF20 [30] (success rate=60%, EF=20.9), whose were validated only for the top 1% ranked molecules for the CASF-2013 dataset. In addition, the results of proposed method are greatly higher than those of the second best performing method GlideScore-SP (with the success rate of 60% and EF of 19%). Additionally, the TopoFormer reaches higher success rates of 81.5% and 87.8% on the top 5% and top 10% ranked molecules. The averaged enrichment factor are 9.7 and 5.6 on top 5% and top 10%, respectively. The AGL-score (success rate=68%, EF=25.6) and ΔVineRF20 (success rate=60%, EF=20.9), were validated only for the top 1% ranked molecules for the CASF-2013 dataset. It also worth to note that some recent deep learning-based models, such as RTMScore [43] (with EF of 28 and success rate of 66.7%), DeepDock [40] (with EF of 16.4 and success rate of 43.9%), and PIGNet [46] (with EF of 19.36 and success rate of 55.4%), but all these model are evaluated on the CASF-2016 core set and trained on different training data, so there is no direct comparison with these method.

As depicted in Figures 3j and 3k, the proposed TopoFormer model surpasses previous methods across both key metrics: success rate and enrichment factor (EF). When tasked with identifying true binders for specific proteins, TopoFormer demonstrates a remarkable success rate of 68% and an average enhancement factor (EF) of 29.6% for the top 1%-ranked molecules. TopoFormer’s results significantly outshine those of the second-best performing method, GlideScore-SP (success rate of 60% and EF of 19%). Furthermore, TopoFormer exhibits high success rates of 81.5% and 87.8% for the top 5% and top 10% ranked molecules, respectively. The corresponding averaged enrichment factors are 9.7 and 5.6 for the top 5% and top 10%, which are the highest performance as shown in Figure 3k. Notably, AGL-score [27] (success rate=68%, EF=25.6) and ΔVineRF20 [30] (success rate=60%, EF=20.9) were assessed solely for the top 1% ranked molecules on CASF-2013 dataset core set. It’s worth highlighting some recent deep learning-based models, including RTMScore [43] (success rate of 66.7% and EF of 28), DeepDock [40] (success rate of 43.9% and EF of 16.4), and PIGNet [46] (success rate of 55.4% and EF of 19.36). However, it is important to note that these models were evaluated on the CASF-2016 core set and trained on different datasets, making direct comparisons with our method impractical.

To understand which scales of the protein-ligand interactions have the most significant impact on the model’s predictions, the saliency map is generated from finetuned TopoFormer_*s*_ for a given protein-ligand complex (PDBID:1E66), as shown in Figure 3h. Specifically, the protein atom within 12 Å around the ligand is considered in the analysis. As suggested from the Figure 3i, the *y*-axis corresponding to different element-specific combination of the given complex, and the *x*-axis is the filtration parameter from 2Å to 12Å. The color bar indicates the gradient on each feature of the topological embedding. The gradients which are significantly higher than the others have been marked with black area, and it denotes the filtration parameter around 4 Å. The saliency map provides insight into the model’s decision-making process by highlighting the relative importance of the topological embedding features at various scales. That means the heavy-atom protein-ligand interaction around scale 4 Å has a stronger influence on the TopoFormer_*s*_ output in the screening task, which is reasonable as hydrogen atoms are not presented in the PDBbind database and our models.

In order to discern the critical facets of protein-ligand interactions that wield the most profound influence on model’s predictions, we have employed the generation of a saliency map with the fine-tuned TopoFormer_*s*_ for a specific protein-ligand complex (PDBID: 1E66), as illustrated in Figure 3h. Our analysis focuses specifically on protein atoms located within a 12 Å radius around the ligand. As depicted in Figure 3i, the *y*-axis corresponds to different element-specific combinations within the given complex, while the *x*-axis represents the filtration parameter ranging from 2 Å to 12 Å. The color bar visually signifies the gradient assigned to each feature within the topological embedding. Distinctive gradients, significantly elevated compared to others, are demarcated by black regions on the map, specifically around the scale of 4 Å in the filtration parameter. The saliency map serves as an invaluable tool for gaining insights into the decision-making process of our model. It accomplishes this by accentuating the relative importance of topological sequence embeddings at various scales. Consequently, it becomes evident that the heavy-atom protein-ligand interactions occurring at approximately 4 Å radius exert a more substantial influence on the output of TopoFormer_*s*_ in the screening task.

## 3 Discussion

In this section, we aim to unravel the intricate web of insights that the TopoFormer brings to the realm of protien-ligand interactions. At its core, the model harnesses the power of persistent topological hyperdigraph Laplacian features, a strategic choice that imbues our framework with a unique prowess in deciphering interaction landscapes.

**Figure 4:**
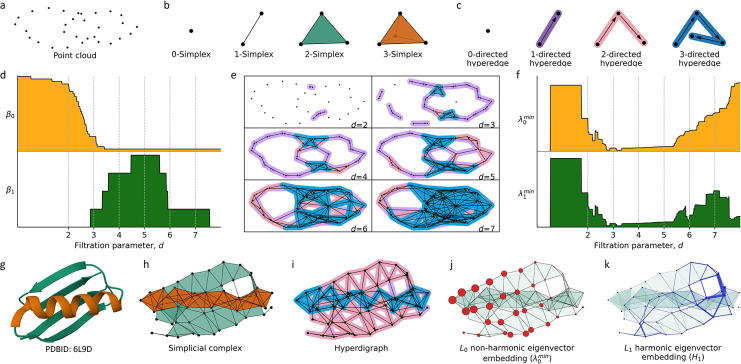
Illustration of the concepts related to topological sequence embedding. **a**, Representation of structural data as a point cloud. **b**, Depiction of 0-simplex (node), 1-simplex (edge), 2-simplex (triangle), and 3-simplex (tetrahedron), which serve as the fundamental building blocks of a simplicial complex. **c**, Illustration of 0-directed hyperedge, 1-directed hyperedge, 2-directed hyperedge, and 3-directed hyperedge, which form the basic building blocks of a hyperdigraph. **d**, Visualization of the multiplicity of zero spectra, i.e., topological invariants, of the persistent topological hyperdigraph at the 0th $\left(\beta_{0}\right)$ and 1st $\left(\beta_{1}\right)$ dimensions, respectively, showcasing their variations with respect to the filtration (scale)parameter d. **e**, Illustration of the impact of varying the filtration parameter on multiscale analysis, resulting in changes in the connectivity of the point cloud and the creation of a sequence of hyperdigraphs, representing a series of topological structures. **f**, Representation of nonzero minimum non-harmonic spectra of the persistent topological hyperdigraph Laplacian at the 0th and 1st dimensions ($\lambda_{0}^{min}$ and $\lambda_{1}^{min}$), highlighting their dependence on the filtration parameter d. **g**, Visualization of protein 6L9D with a representation featuring only $C_{\alpha }$ atoms. The alpha helix is highlighted in orange, while the beta helix is shown in green. **h**, Illustrations of simplicial complex representation for the $C_{\alpha }$ atoms of protein 6L9D at a cutoff distance of d=5Å. The 2-simplices are filled by green, 3-simplices are colored by orange. **i**, Visualizations of hyperdigraph representations for the $C_{\alpha }$ atoms of protein 6L9D at a cutoff distance of d=5Å. The 1-directed hyperedges are depicted as purple edges with arrows, the 2-directed hyperedges are represented by pink edges with arrows, and the 3-directed hyperedges are illustrated as blue edges with arrows. **j**, Description of the $L_{0}$ nonzero minimum non-harmonic eigenvector embedding for the $C_{\alpha }$ atoms of protein 6L9D at a cutoff distance of d=5Å. **k**, Explanation of the $L_{1}$ harmonic eigenvector embedding for the edges between the $C_{\alpha }$ atoms of protein 6L9D at a cutoff distance of d=5Å.

In this study, we employ the persistent topological hyperdigraph Laplacian to give a comprehensive representation for 3D protein-ligand complexes, surpassing traditional graph, simplicial complex, and hypergraph structures (refer to Figure 13). The topological hyperdigraph naturally captures higher-order relationships by allowing directed hyperedges to connect vertices with specific orders, as illustrated in Figure 4c. These directed hyperedges, spanning 0 to 3 dimensions, offer a flexible framework for modeling intricate interactions in protein-ligand complexes, accommodating relationships beyond pairwise connections. By employing directed hyperedges of varying dimensions, our approach provides a nuanced representation of the system’s underlying structure. Additionally, introducing orientations enables encoding of physical/chemical knowledge into directed hyperedges, reflecting differences in electronegativity, atomic radius, weights, and ionization energy for distinct elements. This enhancement serves as an improvement over traditional graph, simplicial complex, and hypergraph representations. Figures 14 g and h showcase hyperdigraph representations for a multi-elemental system, specifically two B_7_C_2_H_9_ isomers, highlighting the capacity to capture different elemental configurations through the directionality of corresponding directed hyperedges.

In the investigation of protein-ligand complexes, we introduce the use of topological hyperdigraphs as an initial step to represent these intricate molecular systems. Subsequently, we incorporate the persistent topological hyperdigraph Laplacian theory [20], to establish a robust and comprehensive framework for analyzing the geometric and topological characteristics of protein-ligand complex systems. Drawing inspiration from physical systems like molecular structures, where the zeroth-dimensional Laplacian matrix is linked to the kinetic energy operator in the Hamiltonian in quantum mechanics [47], we extend this analogy to topological hyperdigraphs. The Laplacian energy, associated with the eigenvalues of the Laplacian matrix in a hyperdigraph context, becomes a valuable tool. These Laplacian eigenvalues offer insights into various properties of the topological object, bearing connections to the energy spectrum of physical systems. Notably, our proposed topological Laplacian analysis in this work provides a means to elucidate the structural and energetic characteristics of complex systems, aligning with fundamental principles in physical systems.

Moreover, in comparison to traditional persistent homology theory, the proposed persistent topological hyperdigraph Laplacian presents significant advancements on multiple fronts. Firstly, it has been demonstrated to effectively analyze the topological hyperdigraph, a high-level generalization encompassing traditional graphs, digraphs, simplicial complexes, and hypergraphs, surpassing the limited applicability of traditional persistent homology theory, which is confined to simplicial complexes. Secondly, the persistent topological hyperdigraph Laplacian provides a more comprehensive approach to characterize protein-ligand complexes. It not only encapsulates the fundamental homology information, such as Betti numbers representing connected components, loops, voids, and higher-dimensional features, but also incorporates additional geometric insights and homotopic shape evolution derived from the non-harmonic spectra of the persistent Laplacians. Illustrated in Figure 10, panels **a**-**e** depict the results of persistent topological hyperdigraph Laplacian analysis for the protein-ligand complex, contrasting with traditional homology analysis in panel **f**. Importantly, it has been confirmed that the multiplicity of zero eigenvalues of the Laplacians corresponds to the Betti numbers, indicating that the barcode information in Figure 10f is encompassed by persistent topological hyperdigraph Laplacians [20], exemplified in Figure 10e.

Considering the diverse scales at which atomic interactions unfold—encompassing phenomena like covalent, ionic, dipole-dipole, and Van der Waals interactions-it becomes apparent that a comprehensive analysis is vital. The proposed persistent topological hyperdigraph Laplacian introduces persistence, offering a multiscale examination of the system. This manifests as a topological sequence evolving with changing in scales, i.e., filtration parameters in the algebraic topology sense, effectively capturing interactions across various scales. This approach proves invaluable in guiding transformer models to discern the distinct contributions of each scale to the desired property, such as binding affinity in protein-ligand complexes, throughout the fine-tuning process. Illustrated in Figures 3 b to e are visualizations depicting the contribution of different scales, i.e., attention scores, for diverse protein-ligand complexes.

Within physical systems, such as the protein-ligand complexes explored in this study [16, 48, 49], a myriad of elemental interactions intricately governs molecular stability and specificity. Hydrogen bonding, van der Waals forces, ionic and polar interactions, nonpolar hydrophobic forces, as well as pi-stacking and dipole-dipole interactions collaboratively mold the structural integrity of the complex. These diverse interactions play pivotal roles in substrate recognition, stability, and the overall specificity of binding events. Recognizing the significance of elemental-level interactions is crucial for deciphering molecular recognition mechanisms, shaping drug design strategies, and advancing our understanding of complex biological processes. To incorporate the elemental interactions between proteins and ligands, we introduce an element-specific analysis, as illustrated in the element-specific hyperdigraph Laplacians module within the topological sequence embedding (Figure 1b). Specifically, interactions between proteins and ligands are considered by constructing sets of common heavy elements in proteins (4 types) and ligands (9 types). Sub-hyperdigraphs of the overall protein-ligand hyperdigraph are generated based on different combinations of these elemental sets, leading to the construction of element-specific Laplacian matrices for each sub-hyperdigraph. The analysis of these matrices encodes the elemental-level interactions within the protein-ligand complex. This element-specific technique enhances the extraction of richer physical and chemical features, aiding the transformer model in comprehending the intricate internal dynamics of protein-ligand complexes under both self-supervised and supervised learning paradigms. More details about the element-specific analysis can be found in the Method Section 4.

## 4 Methods

### 4.1 Datasets

The dataset utilized for pre-training in this study is a comprehensive compilation of protein-ligand complexes (without the labels) sourced from the diverse PDBbind database, including CASF-2007, CASF-2013, CASF-2016, and PDBbind v2020 [39]. To ensure the dataset’s integrity and to eliminate redundancies, a rigorous curation process was meticulously conducted, resulting in a total of 19,513 non-overlapping complexes for pre-training. Rigorous training-test splitting is employed and advocated in this work. For the standard scoring and ranking tasks, the training set comprises the defined refine set, excluding the core set, from PDBbind CASF-2007 (equivalent to PDBbind v2007), CASF-2013 (equivalent to PDBbind v2013), CASF-2016, and PDBbind v2016 datasets. The test set encompasses the respective core sets of these datasets. Given the absence of a core set in PDBbind v2020, the general set (19443), excluding the all core sets from CASF-2007, CASF-2013, CASF-2016, and PDBbind v2016, is employed as the training set (18,904) for the large TopoFormer model. This approach enables a meaningful comparison with recently developed models that have been trained using different data sources. Further details regarding the datasets can be found in Table 2.

For the docking task, the test sets were sourced from the benchmark datasets CASF-2007 and CASF-2013. Each of these datasets consists of 195 test ligands, and for each ligand, 100 poses are generated using various docking programs [25, 24]. In preparation for the docking task training set, a set of 1000 training poses are generated for each given target ligand-receptor pair within the test set. These training poses were generated using GOLD v5.6.33 [50]. Consequently, for both CASF-2007 and CASF-2013, there was a total of 365,000 training poses available for fine-tuning purposes. The pose structures and their corresponding scores, as reported by GOLD, are accessible at https://weilab.math.msu.edu/AGL-Score.

For the screening task, the core set of CASF-2013 was utilized as the test dataset. This set comprises 65 proteins, and each protein interacts with three true binders selected from the 195 ligands within the core set [24]. Regarding the training set, for each target protein present in the test set, the training dataset was constructed using all complex structures and their associated energy labels from the PDBbind v2015 refine set. Notably, the core (test) set complexes were excluded from this training dataset. To augment the training dataset, additional poses and their corresponding labels were generated [45, 27]. It is worth mentioning that the list of true binders for each protein is available in the CASF 2013 benchmark dataset. For each ligand, the pose with the highest energy was used as the upper bound for the training set. All pose structures and their scores can be accessed at https://weilab.math.msu.edu/AGL-Score.

### 4.2 Topological sequence embedding

#### Topological Hyperdigraph.

The topological hyperdigraph serves as a versatile generalization, encompassing digraphs, simplicial complexes, and hypergraphs. It excels in representing intricate relationships such as multi-source to multi-target mappings and asymmetric connections, which are challenging to convey within traditional graphs or simplicial complexes [20]. In essence, the topological hyperdigraph consists of sequences of distinct elements within a finite set, known as directed hyperedges, acting as the fundamental building blocks. Figure 4c provides examples of 0-directed, 1-directed, 2-directed, and 3-directed hyperedges. Notably, these sequences bear a resemblance to the simplices in a simplicial complex. Figure 4b illustrates the 0-simplex (a node), 1-simplex (line segment), 2-simplex (a triangle), and 3-simplex (a tetrahedron) for comparison. For a more in-depth understanding of commonly used graph, simplicial complex, and hypergraph definitions, refer to the Supplementary Information in Section A.3.

A *hyperdigraph* $\vec{ℋ}$ consists of a vertex set V and a collection of sequences with distinct elements in V. An sequence of length k+1 in $\vec{ℋ}$ is called a k-directed hyperedge. Mathematically, a k-directed hyperedge is an inclusion map e:[k]→V, here [k]={0,1,…,k}. A hyperdigraph is a collection of directed hyperedges on V. Sometimes, we denote $\vec{ℋ}=(V,\vec{E})$, where $\vec{E}$ is the set of directed hyperedges. In particular, if the set V is an ordered set, and all directed hyperedges are ordered, then the hyperdigraph can be reduced to a hypergraph. If all directed edges are restricted to be one-dimensional, hyperdigraphs can be simplified to the usual directed graphs. In this sense, hyperdigraphs act more like a versatile aggregator, offering a more flexible and diverse portrayal of data.

More formally, let $C_{k}(V;G)$ be the abelian group generated by the sequences with (k+1) distinct elements in V. Then $C_{*}(V;G)$ is a chain complex with the boundary operator $\partial_{k}\;:C_{k}(V;G)\to C_{k-1}(V;G)$ given by

(1) $$d_{k}\left(x_{0},x_{1},\ldots ,x_{k}\right)=\sum_{i=0}^{k}{\left(-1\right)}^{k}\left(x_{0},\ldots ,{\hat{x}}_{i},\ldots ,x_{k}\right).$$

Here, $\hat{x_{i}}$ means omission of the term $x_{i}$. Let $F_{k}(\vec{ℋ};G)$ be the abelian group generated by the k-directed hyperedges on $\vec{ℋ}$. It follows that $F_{k}(\vec{ℋ};G)$ is a graded subgroup of $C_{*}(V;G)$. We denote

(2) $$\Omega_{k}(\vec{ℋ};G)=\{x\in F_{k}(\vec{ℋ};G)|\partial_{k}x\in F_{k-1}(\vec{ℋ};G)\}.$$

Then, $\Omega_{k}(\vec{ℋ};G)$ is also a chain complex, specifically tailored for exploring the topology of hyperdigraphs. It is essential to highlight that the chain complex $\Omega_{k}(\vec{ℋ};G)$ undergoes simplification when the hyperdigraph is transformed back into a simplicial complex or hypergraph. The corresponding simplicial complex representation of $C_{\alpha }$ atoms in protein 6L9D is depicted in Figure 4h. Here, blue triangles represent the 2-simplices, while orange highlights designate the 3-simplices, providing a rough visualization of the alpha helix structures. Additionally, Figure 4i illustrates the 3-directed hyperedges within the hyperdigraph, highlighted in blue, serving as an alternative representation of the alpha helix in the structure. Figure 13 further presents diverse topological representations, encompassing graphs, simplicial complexes, hypergraphs, and hyperdigraphs. More detailed descriptions and definitions of graphs, simplicial complexes, and hypergraphs are available in the Supplementary Information (see Section A.3) and the original paper [20].

#### Vietoris-Rips hyperdigraph and alpha hyperdigraph.

The Vietoris-Rips (VR) complex and the alpha complex stand out as the most popular topological models for characterizing sets of data points. In the case of 𝒦 being a VR complex or an alpha complex, the points forming a simplex in 𝒦 inherently carry geometric information, encompassing both the magnitude and orientation of the point set. Motivated by the definitions of the VR complex and alpha complex, we introduce the Vietoris-Rips (VR) hyperdigraph and alpha hyperdigraph to capture such geometric information. We employ a weight function w:𝒦→R and a graded orientation function $\varrho_{n}\;:{𝒦}_{n}\to S_{n+1}$ for n≥1 to articulate the geometry of simplices. Here, $S_{n}$ denotes the permutation group of n elements. The VR/alpha hyperdigraph is defined as

(3) $${\vec{ℋ}}_{\eta }\;:=\left\{S\times \varrho_{*}(S)|w(S)\leq \eta ,S\in 𝒦\right\}.$$

The VR/alpha hyperdigraph can be regarded as a generalization of the VR/alpha complex. It simplifies to the VR/alpha complex when assuming the functions w:𝒦→R and $\varrho_{n}\;:{𝒦}_{n}\to S_{n+1}$ are constant.

In this work, all analyses are derived from the VR hyperdigraph without specified instructions. The demonstrations of VR/alpha hyperdigraphs can be found in Figures 5 and 6. The detailed constructions of VR hyperdigraphs and alpha hyperdigraphs are provided in the Supplementary Information A.4.

#### Topological Laplacians and spectrum analysis

The combinatorial Laplacian is a fundamental tool in discrete geometry and algebraic topology. It offers a way to understand the structure of the topological system, such as simplicial complexes, hypergraphs, and hyperdigraphs. Just as the graph Laplacian can be used to study properties of graphs (the graph can be regard as 1-simplices), the combinatorial Laplacian can be used to study properties of simplicial complexes and hyperdigraphs. The eigenvalues of the graph Laplacian can translate the connectivity information of the graph. For example, the second smallest eigenvalue, also known as the Fiedler vector, reflects the algebraic connectivity of the graph, and the smallest positive eigenvalue, also known as the spectral gap, is closely related to the Cheeger constant. The collection of eigenvalues for the Laplacian operator is the spectrum.

Recall that the Laplacian matrix of a graph is given by ℒ=D-A, where D is the degree matrix and A is the adjacency matrix. On the other hand, if the graph is regard as a 1-dimensional simplicial complex, and denote the matrix representing the one-dimensional boundary operator as $B_{1}$, we observe that the Laplacian matrix of the graph can be precisely expressed as $ℒ=B_{1}B_{1}^{T}$. This inspires the generalization of the Laplacian operator to higher dimensions using the boundary operator, leading to the Laplacian operator on simplicial complexes. Let K be a simplicial complex, and let $B_{k}$ be the representation matrix of its k-dimensional boundary operator. The Laplacian matrix is defined as

(4) $${ℒ}_{k}=B_{k+1}B_{k+1}^{T}+B_{k}^{T}B_{k}.$$

Here, $B_{k}^{T}$ denotes the transpose matrix of $B_{k}$. The term $B_{k}^{T}B_{k}$ indicates the connectivity arising from the intersections of k-simplices at (k-1)-simplices, while the term $B_{k+1}B_{k+1}^{T}$ implies the interactions resulting from the inclusions of k-simplices into (k+1)-simplices.

Recall that the topological information for simplicial complexes, hypergraphs, or hyperdigraphs is derived from their respective chain complexes. From now on, we will define the Laplacian operator starting from the perspective of chain complexes. Let $\Omega_{*}$ be a chain complex with the differential $\partial_{k}\;:\Omega_{k}\to \Omega_{k-1}$. Assume that, for each k, there is always an inner product structure on $\Omega_{k}$. Consequently, the boundary operator $\partial_{k}$ has its adjoint operator $\partial_{k}^{*}$. The *combinatorial Laplacian* $\Delta_{k}:\;\Omega_{k}\to \Omega_{k}$ is defined by

(5) $$\Delta_{k}=\partial_{k+1}\circ \partial_{k+1}^{*}+\partial_{k}^{*}\circ \partial_{k}.$$

In particular, $\Delta_{0}=\partial_{1}\circ \partial_{1}^{*}$. For each k, choose a standard orthonormal basis for $\Omega_{k}$, then representation matrix $L_{k}$ of the Laplacian operator $\Delta_{k}$ with respect to the standard orthonormal basis is given by

(6) $${ℒ}_{k}=B_{k+1}B_{k+1}^{T}+B_{k}^{T}B_{k},$$

where $B_{k}$ is the representation matrix of boundary operator $\partial_{k}$ by left multiplication [51]. This combinatorial Laplacian is a generalization of the graph Lapalcian, which is just a carve-out of the properties of graphs (i.e., 1-simplical complex). The combinatorial Laplacian, on the other hand, extends the analysis to higher dimensions. Its eigenvectors and eigenvalues encode geometric and topological information about the simplicial complex or hyperdigraph. Because the Laplacian matrix is positive semidefinite, all eigenvalues of the Laplacian matrix are non-negative. Particularly, the zero eigenvalues, i.e., the harmonic spectrum, encode the topological information. While the non-zero eigenvalues (the non-harmonic spectrum) encode the geometric information about the system. Figure 4j shows the $L_{0}$ nonzero minimum non-harmonic eigenvector embedding for the $C_{\alpha }$ atoms (i.e., 0-simplex in simplicial complex) of protein 6L9D at a cutoff distance of d=5Å. And Figure 4k shows the $L_{1}$ harmonic eigenvector embedding for the edges (i.e., 1-simplex in simplcial complex) between the $C_{\alpha }$ atoms of protein 6L9D at a cutoff distance of d=5Å. Specifically, for ${ℒ}_{k}$, the multiplicity of the zero eigenvalue (i.e., the number of times 0 appears as an eigenvalue) equals the number of independent components, it also equals the topological invariants $\left(\beta_{k}\right)$ in the k-dimensional space [52]. For example, multiplicity of zero for ${ℒ}_{0}$ (i.e., $\beta_{0}$ ) is the number of connected components in the graph (1-simplcial complex), the multiplicity of zero for ${ℒ}_{1}$ (i.e., $\beta_{1}$) is the number of cycles, and it means the number of cavities for ${ℒ}_{2}$. The largest eigenvalue $\lambda_{k}^{max}$ of ${ℒ}_{k}$ is less than or equal to the maximum number $d_{k}$ of k+1-simplex shared one k-simplex (maximum degree of the graph for ${ℒ}_{0}$). Specifically, $0\leq \lambda_{k}^{max}\leq 2d_{k}$. The smallest non-zero eigenvalue for ${ℒ}_{k}$, also know as spectral gap, denoted as $\lambda_{k}^{min}$, reflect the geometric structure of the system. In this work, the multiplicity of zero, the average value, the standard deviation, the minimum, the maximum, and the summation of the positive eigenvalue for ${ℒ}_{0}$ are used to embed the given topological Laplacians. In addition, to validate the power of topological hyperdigraph Laplacian, two B_7_C_2_H_9_ isomers with identical geometric structures, differing only in the positions of carbon atoms are constructed in the validation, as shown in Figure 14. The findings indicate that the hyperdigraph Laplacian possesses the capacity to encode more information compared to standard Laplacians.

#### Persistent Laplacians

Persistent Laplacians or multiscale topological Laplacians, were introduced in a series of papers on a differential manifold setting [53] and a discrete point cloud setting [18, 54] in 2019. A filtration process is essential to achieving the multiscale representation in persistent Laplacians [18, 20, 55] as well as in persistent homology [21, 56]. The choice of the filtration (scale) parameter, denoted as d, varies based on the data structure in question: for point cloud data (Figure 4a), it is often the sphere radius (or diameter). By systematically adjusting d, one can derive a sequence of hierarchical representations, illustrated in Figure 1a. Notably, these representations are not limited to simplicial complexes, but can also be realized with hyperdigraphs. As an example, consider a filtration operation applied to a distance matrix, where the matrix elements represent distances between vertices. One could define a cutoff value as the scale parameter; if the distance between two vertices falls below this cutoff, they are connected. By progressively increasing this cutoff, one obtains a sequence of nested graphs. Each graph in this sequence, derived from a smaller cutoff value, is a subset of the graph generated with a higher cutoff.

In a similar vein, nested simplicial complexes can be formed based on different complex definitions like the Vietoris-Rips complex, Čech complex, and alpha complex. The Vietoris-Rips complex is used in this work. Mathematically, the nested simplicial complexes can be written as:

(7) $$\emptyset \subseteq K_{d_{0}}\subseteq K_{d_{1}}\subseteq \cdots \subseteq K_{d_{n}}=K$$

Here, for any two $d_{i}<d_{j}$, we have $K_{d_{i}}\subseteq K_{d_{j}}$. The concept extends to hyperdigraphs as well, namely Vietoris-Rips hyperdigraph: one can form nested hyperdigraphs by properly defining directed hyperedges [20]. To visualize the effects of changing filtration parameters, Figure 4e depicts alterations in point cloud connectivity from Figure 4a, leading to a sequence of hyperdigraphs. Additionally, Figure 11 showcases simplicial complex produced at different filtration parameters and Figure 12 illustrates hyperdigraphs generated at different filtration parameters. The details about the construction of Vietoris-Rips hyperdigraph can be seen in Figure 5. In addition, inspired by the alpha complex, the alpha hyperdigraph is also introduced in this work, as shown in Figure 6.

As a filtration process unfolds, it naturally gives rise to a family of chain complexes. For each filtration step $d_{i}$ (with i indexing the steps), a chain complex $C\left(K_{d_{i}};G\right)$ is constructed. Mathematically, a chain complex for a particular filtration step is a sequence of Abelian groups (or modules) and boundary homomorphisms:

(8) $$\cdots \to C_{k+1}\left(K_{d_{i}};G\right)\overset{\partial_{k+1}^{d_{i}}}{\to }C_{k}\left(K_{d_{i}};G\right)\overset{\partial_{k}^{d_{i}}}{\to }C_{k-1}\left(K_{d_{i}};G\right)\to \cdots$$

where $C_{k}\left(K_{d_{i}};G\right)$ is the k-dimensional chain group at filtration step $d_{i}$.

For a more general exposition, we now introduce the Laplacian in a mathematical formalism. For real numbers a≤b, let $\Omega_{*}^{a}$ and $\Omega_{*}^{b}$ be chain complexes. Suppose that $\Omega_{*}^{a}\subseteq \Omega_{*}^{b}$. The chain complexes considered can be the chain complexes obtain from a filtration of simplicial complexes, hypergraphs, or hyperdigraphs, among other possibilities. Moreover, the chain complexes $\Omega_{*}^{a}$ and $\Omega_{*}^{b}$ are endowed with the compatible inner product structures. Let $\Omega_{k+1}^{a,b}=\{x\in \Omega_{k+1}^{b}|\partial_{k+1}^{b}x\in \Omega_{k}^{a}\}$. The persistent boundary operator $\partial_{k+1}^{a,b}\;:\Omega_{k+1}^{a,b}\to \Omega_{k}^{a}$ is defined by $\partial_{k+1}^{a,b}x=\partial_{k+1}^{b}x$ for $x\in \Omega_{k+1}^{a,b}$.
(9)
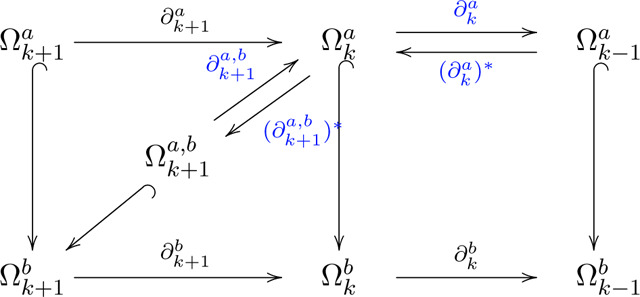

The k-*th persistent Laplacian* is defined as

(10) $$\Delta_{k}^{a,b}=\partial_{k+1}^{a,b}\circ {\left(\partial_{k+1}^{a,b}\right)}^{*}+{\left(\partial_{k}^{a}\right)}^{*}\circ \partial_{k}^{a}.$$

It is worth noting that the harmonic part of $\Delta_{k}^{a,b}$, i.e., ker $\Delta_{k}^{a,b}$, is naturally isomorphic to the (a,b)-persistent homology $H_{k}^{a,b}=im(H_{k}\left(\Omega_{*}^{a}\right)\to H_{k}{\left(\Omega^{b}\right)}_{*})$ [57]. In a broad sense, the harmonic part of the persistent Laplacian contains information about persistent homology. To glean insights from each chain complex, one can resort to spectrum analysis. By constructing the Lapalcian matrices corresponding to each $\partial_{k}$ and $\partial_{k+1}$ and examining their spectra (eigenvalues and eigenvectors), one can uncover rich structural information about the topological and geometric properties inherent in the data at that particular scale of the filtration. This spectral information often provides a compact and informative summary of the data, allowing for efficient comparison and analysis across different scales. Figure 4d illustrates the evolution of zero eigenvalue multiplicities in the associated Laplacian matrix as the filtration (scale) parameters change, while Figure 4f depicts the variation in the minimum positive eigenvalue with changing filtration (scale) parameters. Additional persistent attributes are presented in Figure 10.

#### Element-specific embedding

In this work, the topological embedding method is applied to encoding the protein-ligand complex. An accurate prediction requires a better representation of the interactions between proteins and ligands at the molecular level. Here, the element-specific topological embedding [16] is used to characterize protein-ligand interactions.

When analyzing ligands, the focus is on heavy elements such as carbon (C), nitrogen (N), oxygen (O), sulfur (S), phosphorus (P), fluoride (F), chloride (Cl), bromide (Br), and iodine (I). Conversely, for proteins, only carbon (C), nitrogen (N), oxygen (O), and sulfur (S) are considered. Subsequently, a range of element combinations, arranged in a specific sequence, will represent the interactions between the protein and the ligand. For proteins, the combinations are denoted as ${ℰ}_{\text{protein}}=\{\{C\},\;\{N\},\;\{O\},\;\{S\},\;\{C,\;N\},\;\{C,\;O\},\;\{C,\;S\},\;\{N,\;O\},\;\{N,\;S\},\;\{O,\;S\},\;\{C,\;N,\;O,\;S\}\}$. Meanwhile, the ligand combinations are ${ℰ}_{\text{ligand}}=\{\{C\},\;\{N\},\;\{O\},\;\{S\},\;\{C,\;N\},\;\{C,\;O\},\;\{C,\;S\},\left\{N,\;O\right\},\left\{N,\;S\right\},\left\{O,\;S\right\},\left\{N,\;P\right\},\left\{F,\;Cl,\;Br,\;I\right\},\{C,\;O,\;N,\;S,\;F,\;P,\;Cl,\;Br,\;I\}\}$. Within the Element-specific embedding approach, the interactions between proteins and ligands are defined by the topological links between two sets of atoms: one from the protein and the other from the ligand. For example, a representation like $K_{\left\{C,N\},\{S\right\}}$ indicates the topological hyperdigraph representation where the C and N atoms are derived from the protein, while the S atom comes from the ligand. The Element-specific embeddings detail interactions based on their spatial relationships. It can be characterized by distance matrix D as follows,

(11) $$D(i,j)=\left\{\begin{array}{ll} ∥r_{i}-r_{j}∥, & \text{if}\;r_{i}\in {ℰ}_{\text{protein}},r_{j}\in {ℰ}_{\text{ligand}}\;\text{or}\;r_{i}\in {ℰ}_{\text{ligand}},r_{j}\in {ℰ}_{\text{protein}}\\ \infty , & \text{other} \end{array}\right.$$

where the $r_{i}$ and $r_{j}$ are coordinates for the i th and j th atoms in the set, and $∥r_{i}-r_{j}∥$ is their Euclidean distance. In the TopoFormer model, protein atoms located within 20 Å of ligand atoms are taken into account. For the TopoFormer_*s*_ model, the range is reduced to protein atoms within 12 Å of the ligand atoms. In this study, emphasis is placed on the protein-ligand interactions by assigning an infinite value to the distance between atoms either within the protein or the ligand. For a specific protein-ligand complex, there are 143 potential combinations (derived from 11 protein sets multiplied by 13 ligand sets). Each of these combinations functions as a simplicial complex and is further examined using the persistent topological hyperdigraph Laplacian approach.

### 4.3 TopoFormer model

#### Model architecture.

The TopoFormer model introduced in our work incorporates a Topological embedding model. This model transforms the 3D protein-ligand complex into a topological sequence characterized by topological features at various scales. Specifically, in the larger version of the TopoFormer model, the scale range extends from 0 Å to 10 Å in increments of 0.1 Å, resulting in a topological sequence of 100 units in length. At each filtration (scale) increment, the embedded features possesses a matrix of 143 by 6 (6 attributes associated with each ${ℒ}_{0}$). The combined outputs from the topological embedding module are obtained by summing the topological embeddings with the trainable multiscale embeddings, as depicted in Figure 1a. To convert the 143 by 6 matrix for every filtration increment into a 1-dimensional vector, we have incorporated a convolutional layer into both the Transformer’s original encoder and decoder, as shown in Figure 1c. Subsequently, the conventional dot-product attention mechanism in the Transformer utilizes encoded representations of the input in the form of queries (Q), keys (K), and values (V) designated for each filtration increment. This attention can be mathematically represented as,

(12) $$Attention(Q,K,V)=softmax\left(\frac{QK^{T}}{\sqrt{d_{k}}}\right)V$$

Here, the $\sqrt{d_{k}}$ is the scalar defined by the root of embedding dimension($d_{k}=512$ in this work). The resulting bidirectional attention matrix is then derived from this formula. In addition, similar with the MAE model [58] in computer vision, an asymmetric design is applied for TopoFormer’s encoder and decoder. Detailed settings of the TopoFormer are provided in Supplementary Information Section SA.2. The training process of the model encompasses two phases: initially, self-supervised learning is applied to unlabeled data to obtain a pre-trained model. Subsequently, supervised learning is employed on specific benchmarks tailored to various tasks, resulting in a fine-tuned model.

#### Self-supervised and supervised learning in TopoFormer.

In this study, we utilized 19,513 unlabeled protein-ligand complexes from the PDBbind database for the pretraining of TopoFormer. The topological embeddings derived from these complexes were reconstructed and subsequently employed to compute the reconstruction loss. For this purpose, the mean square error (MAE) was adopted as the metric for reconstruction loss. This self-supervised approach enables the model to discern deep, generalized representations of protein-ligand complex patterns using a vast amount of unlabeled data. Such an approach potentially simplifies the downstream fine-tuning process. In this study, a dataset of only nearly 20,000 unlabeled complexes yielded exceptional performance across most tasks. Moving forward, we envisage incorporating even more protein-ligand complexes into the pretraining workflow, without the necessity for experimental data. In this study, all tasks encompassing scoring, ranking, docking, and screening involve fine-tuning the TopoFormer model to predict a specific score for a given protein-ligand complex. Consequently, the mean square error was selected as the loss function for these tasks.

## Supplementary Material

Supplement 1

## Figures and Tables

**Table 1:** The PCCs(RMSE in kcal/mol) of our TopoFormer models on the three benchmarks of CASF-2007, CASF- 2013, and CASF-2016. TopoFormer and TopoFormers are considered. The averages of 400 repetitions are computed as the performance of the model. The detailed setting of two TopoFormers and GBRT parameters can be found in Supplementary Information Section A.2.

Dataset	CASF-2007	CASF-2013	CASF-2016	Average

TopoFormer-Seq	0.837(1.807)	0.816(1.859)	0.864(1.568)	0.839(1.745)
TopoFormer_*s*_-Seq	0.839(1.798)	0.809(1.886)	0.855(1.609)	0.834(1.764)
TopoFormer	0.826(1.830)	0.788(1.910)	0.849(1.595)	0.821(1.778)
TopoFormer_*s*_	0.826(1.832)	0.781(1.944)	0.836(1.657)	0.814(1.811)
Seq-ML	0.798(1.974)	0.790(1.960)	0.837(1.693)	0.808(1.876)

**Table 2:** Detailed information of the used datasets.

	Datasets	Training set	Test set (core set)
Pretraining (Self-supervised Learning)	Combind PDBbind (CASF-2007, CASF-2013, PDBbind v2015, CASF-2016,v2020)	19513	/
Finetuning (Supervised Learning)	CASF-2007	1105	195
	CASF-2013	2764	195
	CASF-2016	3772	285
	PDBbind v2016	3767	290
	PDBbind v2020	18904	195(CASF-2007 core set)
			195(CASF-2013 core set)
			285(CASF-2016 core set)

## Data Availability

The training dataset employed in this study comprises a comprehensive collection of protein-ligand complexes sourced from various PDBbind databases, specifically CASF-2007, CASF-2013, CASF-2016, and PDBbind v2020. To ensure the dataset’s reliability and eliminate redundancies, a meticulous curation process was undertaken, resulting in a total of 19,513 non-overlapping complexes. And all data used in this study can be downloaded from the official PDBbind website: http://www.pdbbind.org.cn/index.php. Additionally, the topological embedded features utilized in both TopoFormer and TopoFormer_*s*_, as well as the sequence-based features derived from the Transformer-CPZ model [22] and the ESM model [31], are readily available for download at https://github.com/WeilabMSU/TopoFormer. The additional generated poses and their associated scores, which were instrumental in the docking and screening tasks, can be obtained from the following source: https://weilab.math.msu.edu/AGL-Score.
