# Supplementary material for: TopoFormer: Multiscale Topology-enabled Structure-to-Sequence Transformer for Protein-Ligand Interaction Predictions
Source: Res Sq. 2024 Feb 9:rs.3.rs-3640878. Preprint. [Version 1] doi: 10.21203/rs.3.rs-3640878/v1 (PMC10889053; doi:10.21203/rs.3.rs-3640878/v1)
Supplement: Supplement 1 [file NIHPPRS3640878V1-supplement-1.pdf]

A Supplementary Information

This document provides additional details not essential to the main body of the paper but potentially of interest to readers.

A.1 Evaluation metrics

Evaluation of scoring power. In this study, the Pearson correlation coefficient (PCC) is used in the evaluation of scoring power, and it is defined as below:

$$\text{PCC} = \frac{\sum (x_i - \bar{x})(y_i - \bar{y})}{\sqrt{\sum (x_i - \bar{x})^2 \sum (y_i - \bar{y})^2}} \tag{13}$$

where x_i is the value of the x variable in ith sample, $\bar{x}$ is the mean of the values of the x variable, y_i is the value of the y variable in the ith sample, $\bar{y}$ is mean of the values of the y variable. The Pearson correlation coefficient (PCC) explains the relationship between the x variable and y variable.

The root mean squared error (RMSE) is defined as below:

$$\text{RMSE} = \sqrt{\frac{1}{n}\sum_{i=1}^{n}(y_i - \hat{y}_i)^2} \tag{14}$$

where y_i and $\hat{y}_i$ are predicted value and true value of ith sample respectively.

Evaluation of ranking power. In this work, two evaluative approaches are employed: the high-level and the low-level success measurements. In the high-level success metric, the objective is to perfectly rank the binding affinities of the complexes within each cluster. Conversely, the low-level success criterion requires the scoring function to merely identify the complex with the pinnacle binding affinity. The assessment of ranking efficacy termed "ranking power" is gauged by the proportion of correctly identified affinities across a specified benchmark.

Let us denote the set of protein-ligand complexes in a given cluster as C, and let A_i be the binding affinity of the ith complex in C, where smaller i indicates smaller binding affinity. For a cluster C with n complexes ($n = 3$ for benchmarks CASF-2007 and CASF-2013, $n = 5$ for CASF-2016): The scoring function f is successful in the sense of high-level if and only if:

$$f(A_i) \geq f(A_j), \text{if } i \geq j \text{ and } A_i, A_j \in C \tag{15}$$

The low-level success measurement is defined as:

$$f(A_{max}) > f(A_i), \forall A_i \in C \tag{16}$$

where A_{max} is the complex with the highest binding affinity in C.

The "Ranking Power" of a scoring function across a benchmark can then be calculated as:

$$\text{Ranking Power} = \frac{\text{Number of successful clusters by the function}}{\text{Total number of clusters in the benchmark}} \times 100\% \tag{17}$$

This provides a percentage-based assessment of how often the scoring function correctly ranks the binding affinities within the given clusters. Notably, the current iteration of the ranking power

metric can be optimized. Presently, it is restricted to determining the accurate binding affinity sequence of three native ligands for each target receptor in the core set. This might not adequately mirror the complexities of an authentic virtual screening scenario where a multitude of ligands might vie for the same target receptor. Incorporating more comprehensive evaluation metrics, like Kendall's tau or the Spearman correlation coefficient, could enhance accuracy. In our study, the results of high level success measurement are shown in Figure S 8, and the low level success measurement are shown in Figure S 9.

Evaluation of docking power. The present assessment evaluates a scoring function's proficiency in distinguishing the "native" pose from an array of poses generated by docking software. Within the benchmark parameters, a pose is deemed "native" if its root-mean-square deviation (RMSD) relative to the genuine binding pose is less than 2 Å. To ensure alignment with prior research, we have anchored our validation efforts to both the CASF-2007 and CASF-2013 datasets, adhering to training and test sets as delineated in the extant literature [27, 25, 24]. In the CASF-2007 benchmark, each ligand was supplied with 100 distinctive poses, all generated using specific docking software packages. Meanwhile, the CASF-2013 benchmark produced 100 poses for each ligand, courtesy of three eminent docking applications: GOLD v5.1, Surflex-Dock (integrated within SYBYL v8.1), and MOE v2011. For researchers seeking accessibility, the curated poses can be procured from https://weilab.math.msu.edu/AGL-Score/. It is worth noting that in both benchmarks, owing to structures that exhibit certain symmetries, a given ligand may possess multiple "native" poses within the dataset. As such, if a method successfully discerns any of these native poses, it is adjudged as successful for that ligand. The ultimate measure of efficacy, termed "docking power", is gauged by the tally of ligands for which "native" poses are accurately pinpointed. It can be calculated as:

$$\text{Docking Power} = \frac{\text{Number of complexes that successfully identified ``native'' poses}}{\text{Total number of complexes in the benchmark}} \times 100\% \quad (18)$$

In the docking task, the Root Mean Square Deviation (RMSD) is a measure used to assess the similarity between the predicted or generated molecular structure (usually a ligand) and a reference structure (often the experimentally determined or known structure). RMSD is often used to evaluate the accuracy of how a docking program predicts the binding mode of a ligand within a protein's binding site. It is defined as:

$$\text{RMSD} = \sqrt{\frac{\sum_i (A_i - B_i)^2}{n}}, \quad (19)$$

where A_i is the coordinates of the i-th atom in the docked structure, B_i is the coordinate of the i-th atom in the experimental structure, and n means the total number of atoms being compared in both structures. RMSD help to determine how well a docking program can reproduce known binding poses or predict the binding mode of a ligand within a protein's active site. Lower RMSD values are generally desirable, indicating more accurate predictions.

Evaluation of screening power. There are two kinds of screening power measurements. The first one the enrichment factor in protein-ligand screening, which is often used in the field of computational chemistry and drug discovery. The enrichment factor (EF) is a measure of how

effectively a virtual screening or docking method enriches active or potent compounds (ligands) within a larger library of compounds. It is used to assess the performance of these methods in identifying potential drug candidates. The enrichment factor is typically calculated as follows

$$\mathrm{EF} = \frac{\text{Number of true positives}}{\text{Number of total hits}} \cdot \frac{\text{Total number of compounds}}{\text{Number of active compounds}}, \tag{20}$$

where the "Number of true positives" is the number of active compounds correctly identified as hits by the TopoFormer model. Number of total hits is the total number of compounds identified as hits by the screening method. Number of active compounds means the total number of active or potent compounds in the entire test set. The total number of compounds means the total number of compounds in the test set. The objective of the second screening power measurement is to pinpoint the optimal true binder. The success rate is determined by the $x\%$ of the top-ranked candidates, among which the best binders from a pool of 65 receptors are discovered.

Loss function for training. In this work, the mean squared error (MSE) is applied as the loss function during the pre-training and fine-tuning stages. MSE is a widely used metric to quantify the difference between predicted and actual values in statistical modeling and machine learning. It measures the average squared differences between predictions and actual observations. The mathematical definition of MSE is:

$$\mathrm{MSE} = \frac{1}{n}\sum_{i=1}^{n}(y_i - \hat{y}_i)^2 \tag{21}$$

where y_i is the actual value for the i-th data point. $\hat{y}_i$ is the predicted value for the i-th data point. And n is the total number of data points.

A.2 Hyperparameter selection and optimization

In the Seq-ML approach, we employ the Gradient Boosted Decision Trees (GBDT) algorithm to predict protein-ligand binding affinity. The parameters are set as follows: 'n_estimators' to 10,000, 'max_depth' to 7, 'min_samples_split' to 2, with a subsample size of 0.4, and a learning rate set at 0.005. All other parameters retain their default values as defined in the algorithm [59]. For the classification task within the screening process, the GBDT parameters remain consistent with those described for the regression task.

In the TopoFormer models, we utilize a self-supervised learning approach in the pre-training phase, followed by a supervised learning strategy during the fine-tuning stage. Relevant parameters are detailed in Table 3.

In the pre-training stage of our proposed model, we diligently selected training hyperparameters to encourage robust learning and convergence. A batch size of 64 and a maximum of 30,000 training steps were utilized, alongside an initial learning rate of 0.001, ensuring a smooth and steady journey towards optimal weight adjustments. A warm-up period, comprising 5% of the maximum training steps, was integrated to stabilize the initial training phase, incrementally increasing the learning rate from 0 to the set initial rate. Following this, the fine-tuning stage implemented a supervised learning strategy, ensuring task-specific model refinement without overfitting the hyperparameters. The batch size was reduced to 32 and the initial learning rate was slightly diminished to 0.0008. Distinct

Table 3: The parameter settings for TopoFormer

Parameters	Pre-training stage	Finetuning stage
attention_probs_dropout_prob	0.1	0.1
decoder_hidden_size	768	/
decoder_intermediate_size	3072	/
decoder_num_attention_heads	12	/
decoder_num_hidden_layers	8	/
hidden_act	gelu	gelu
hidden_dropout_prob	0.1	0.1
hidden_size	1024	1024
image_size(large)	(100, 143)	(100, 143)
image_size(small)	(50, 143)	(50, 143)
initializer_range	0.02	0.02
intermediate_size	4096	4096
num_attention_heads	4096	4096
num_channels	6	6
num_hidden_layers	12	12
patch_size	(1, 143)	(1, 143)

maximum training steps were employed for varying tasks: 10,000 steps for scoring tasks, and a more succinct 5,000 steps for both the docking and screening tasks. It is noteworthy that specific parameters, such as the warm-up steps and optimizer, were consistently held across both pre-training and fine-tuning stages, ensuring a coherent model development. Furthermore, for the fine-tuning of the scoring task, additional parameter combinations proximate to the pre-defined settings were tested to validate the robustness of the proposed model. Specifically, combinations of batch size 64 with a learning rate of 0.0008, and batch size 32 with a learning rate of 0.001 were examined. The results, delineated in Table 4, reveal closely tied performances across the different settings, underscoring the model's stability and robustness amidst variations in the hyperparameters.

A.3 Topological objects

Graph. Graph is the most fundamental object for describing relationships among entities and is one of the most common data types. It consists of nodes and edges, capturing the relationships between nodes. Common extensions of graphs include directed graphs, weighted graphs, and geometric graphs, among others. These graph-based models often provide an effective representation of relationships and characteristics within various contexts. Strictly speaking, a *graph* is a pair (V, E), where V is a vertex set and $E \subseteq V \times V$ is the edge set. Vertices and edges are the fundamental objects of a graph. Various tools are employed to characterize the relationships between points and edges, such as adjacency matrices, degree matrices, and Laplacian matrices. These matrices play a crucial role in graph theory and network analysis, effectively capturing the topological structure of the graph. Given that a graph inherently has a 1-dimensional structure, certain models from simplicial complexes are also employed to capture the higher-dimensional structures of the graph. Examples include the clique complex, neighborhood complex, and Hom complex [60, 61].

Simplicial complex. A simplicial complex is a topological space that is built up from simple pieces called simplices. A simplex is a generalization of the concept of a triangle or tetrahedron to arbitrary dimensions. Given a vertex set V, a k-simplex σ is often represented by a $(k+1)$-element subset of vertices in V, denoted as $\sigma = \langle v_0, v_1, \ldots, v_k \rangle$. And a subset of σ is a face of σ.

A *simplicial complex* K on a vertex set V is a collection of simplices satisfying the following two conditions: (1) If a simplex σ is in K, then so is each face of σ, including the individual vertices; (2) The intersection of any two simplices in K is either an empty set or a face (subset) of both simplices. Using the above properties, it is clear that a graph can be viewed as a 1-dimensional simplicial complex, as its simplices are its vertices (0-simplices) and edges (1-simplices).

For a given k-simplex, the boundary is essentially the collection of its $(k-1)$-dimensional faces. Mathematically, the *boundary operator*, denoted by ∂_k, acts on a k-simplex $\langle v_0, v_1, \ldots, v_k \rangle$ as:

$$\partial_k \langle v_0, v_1, \ldots, v_k \rangle = \sum_{i=0}^{k} (-1)^i \langle v_0, \ldots, \widehat{v_i}, \ldots, v_k \rangle, \tag{22}$$

where $\widehat{v_i}$ means that vertex v_i is omitted. A chain complex is a sequence of Abelian groups (or modules) connected by boundary operators. Let G be an abelian group. The k-th group, denoted as $C_k(K; G)$, in the chain complex consists of formal sums of k-simplices, and the boundary operator $\partial_k : C_k(K; G) \to C_{k-1}(K; G)$ maps a k-simplex to its $(k-1)$-dimensional boundary. The chain complex can be represented as a sequence like this:

$$\cdots \xrightarrow{\partial_{k+1}} C_k(K; G) \xrightarrow{\partial_k} C_{k-1}(K; G) \xrightarrow{\partial_{k-1}} \cdots \xrightarrow{\partial_2} C_1(K; G) \xrightarrow{\partial_1} C_0(K; G). \tag{23}$$

An essential property of the boundary operator is that the composition of two successive boundary operators is zero, i.e., $\partial_{k-1} \circ \partial_k = 0$. It means that the boundary of a boundary is always zero, which has topological implications. The chain complex structure provides a framework to understand how the boundaries fit together.

While simplicial complexes serve as topological models to depict relationships in most data, there are instances where they remain somewhat restrictive. In such cases, topological hypergraphs, as a more general model and combinatorial object, exhibit significant potential in applications.

Topological hypergraph. Topological hypergraph, as a relatively new combinatorial object, can be considered as a generalization of the concepts of graphs and simplicial complexes. From a graph perspective, topological hypergraphs can be seen as an extension of edges in graphs, where edges are not limited to pairs of vertices but can include multiple vertices. From a simplicial complex perspective, topological hypergraphs can be viewed as relaxing the condition that the faces of simplices must be simplices.

A *topological hypergraph* $\mathcal{H}$ on a vertex V is a collection of subsets of V. The $(k+1)$-element subsets of V are the *k-hyperedges*. The *simplicial closure* of a topologcal hypergraph $\mathcal{H}$ is given by

$$\Delta\mathcal{H} = \{\sigma | \sigma \subseteq \tau \text{for some hyperedge } \tau \in \mathcal{H}\}. \quad (24)$$

The simplicial $\Delta\mathcal{H}$ closure is the minimal simplicial complex containing $\mathcal{H}$. In light of the close connection between topological hypergraphs and simplicial complexes, topological hypergraphs can always be constructed based on simplicial complexes, which inspires the study of the topological structures of topological hypergraphs. Recently, embedded homology for topological hypergraphs has been introduced to investigate their topological features [62]. Let $D_k(\mathcal{H}; G)$ be the abelian group generated by the k-hyperedges. Then $D_*(\mathcal{H}; G)$ is a graded subgroup of the chain complex $C_*(\Delta\mathcal{H}; G)$ of the simplicial complex $\Delta\mathcal{H}$. Thus, one can obtain the infimum complex

$$\mathrm{Inf}_*(\mathcal{H}; G) = \{x \in D_*(\mathcal{H}; G) | \partial x \in D_*(\mathcal{H}; G)\}. \quad (25)$$

Here, ∂ is the boundary operator on $C_*(\Delta\mathcal{H}; G)$. The name "infimum complex" primarily stems from the fact that $\mathrm{Inf}_*(\mathcal{H}; G)$ is the minimal sub chain complex of $C_*(\Delta\mathcal{H}; G)$ containing $D_*(\mathcal{H}; G)$. The topological information on hypergraphs is based on the infimum complex $\mathrm{Inf}_*(\mathcal{H}; G)$.

Topological hypergraphs have become a very general research object for studying interactions in complex systems. However, when exploring complex systems and structures involving directional and asymmetric relationships, topological hypergraphs may not be sufficiently inclusive. In such cases, topological hyperdigraphs, as objects incorporating higher-dimensional structures, multi-faceted interactions, and directional information, become our new focus.

A.4 Vietoris-Rips hyperdigraph and alpha hyperdigraph

The Vietoris-Rips (VR) hyperdigraph is constructed based on the VR complex. Let (M, d) be a metric space. Let X be a finite point set in M. For a given parameter d, the VR complex $\mathcal{VR}_d$ is defined by

$$\mathcal{VR}_d = \{S \subseteq X | \text{every two points } x, y \text{ in } S \text{ has the distance } d(x, y) \leq d\}. \quad (26)$$

The VR complex is always regarded as an abstract simplicial complex; that is, a simplex S is considered only as a set, without considering its geometric structure. This provides us with the motivation to study more general structures. If we take into account geometric properties such as angles, volumes, or even their manifestations in biology or materials, then the hyperdigraph becomes a more versatile topological model. Specifically, for any simplex S, we assign to it both weight information and orientation information. Mathematically, for a VR complex $\mathcal{VR}_d$, there is a weight function $w : \mathcal{VR}_d \to \mathbb{R}$ and a graded orientation function $\varrho_n : (\mathcal{VR}_d)_n \to S_{n+1}$ for

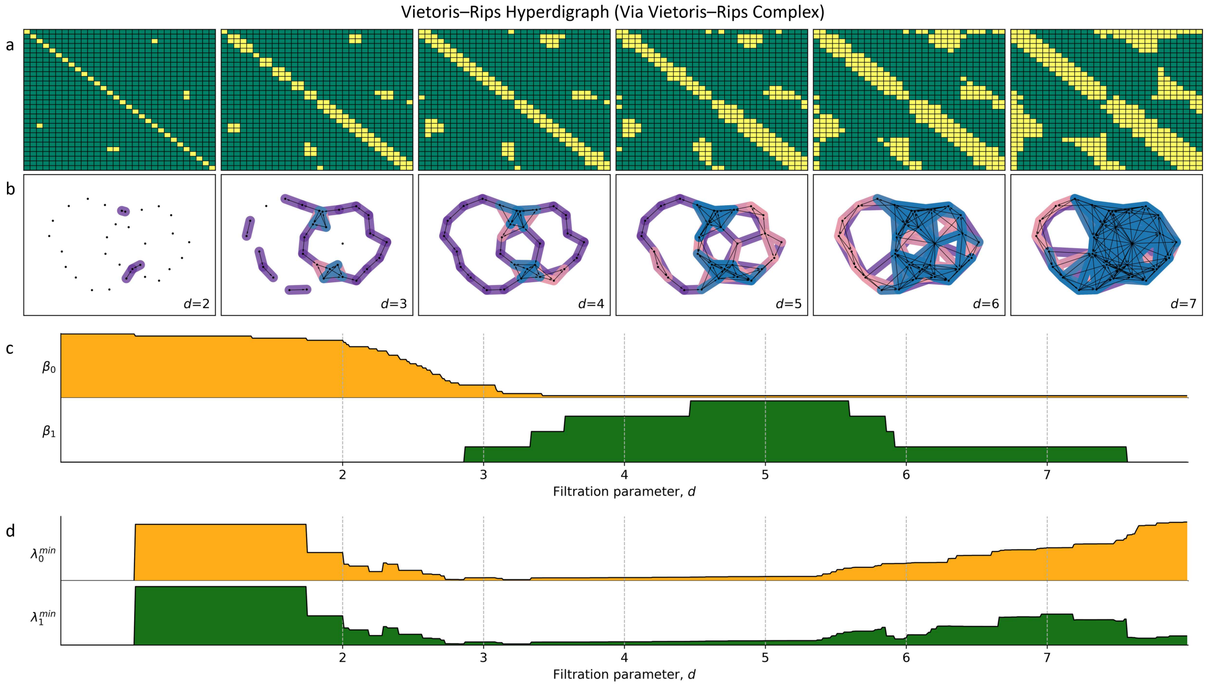

Figure 5: Illustration of Vietoris-Rips hyperdigraph construction with over scales for the point cloud in Figure 4**a**. **a** Illustration of the adjacency matrices of the point cloud at various scales (i.e., filtration parameter d values). Yellow entries in the matrices represent connections between points with distances smaller than the threshold, while green entries indicate points that are not connected. **b** The constructed Vietoris-Rips hyperdigraphs at various scales, including $d = 2$, $d = 3$, $d = 4$, $d = 5$, $d = 6$, and $d = 7$. **c** A display of the persistent Betti numbers, denoted as β_i with $i = 0$ and $i = 1$. Vertical dashed lines mark the Betti numbers corresponding to specific scales. **d** The nonzero minimum non-harmonic spectra of the persistent topological hyperdigraph Laplacian at the 0th and 1st dimensions (λ_0^{min} and λ_1^{min}), highlighting their dependence on the scale parameter d.

$n \geq 1$. Here, S_n is the permutation group of n elements. Then for any $\eta \in \mathbb{R}$, the *Vietoris-Rips hyperdigraph* is defined by

$$\mathcal{VR}_d\vec{\mathcal{H}}_\eta := \{S \times \varrho_*(S) | w(S) \leq \eta, S \in \mathcal{VR}_d\}. \quad (27)$$

Note that there is a one-one corresponding between the sequences and the permutation group for a fixed length [20]. Thus the element $S \times \varrho_*(S)$ is essentially a sequence. The homology and Laplacians of hyperdigraphs can be computed to detect topological and geometric features of point set. In this work, the weight function $w : \mathcal{VR}_d \to \mathbb{R}$ and the graded orientation function $\varrho_n : (\mathcal{VR}_d)_n \to S_{n+1}$ are taken to be the trivial functions. Consequently, the hyperdigraph construction can be simplified to coincide with the Vietoris-Rips complex.

Informally, a Vietoris-Rips (VR) complex is a simplicial complex whose simplices are formed by finite sets of points, with the condition that any two points in the set are no larger than a specified threshold parameter, known as the filtration parameter. Subsequently, directed hyperedges are constructed on all simplices in the complex. In this process, we take into account the geometric information of the simplices, considering both their direction and magnitude. By selecting certain simplices and endowing them with orientations, we form a collection of selected simplices, constituting a hyperdigraph. If the direction for each directed hyperedge is determined by a pre-

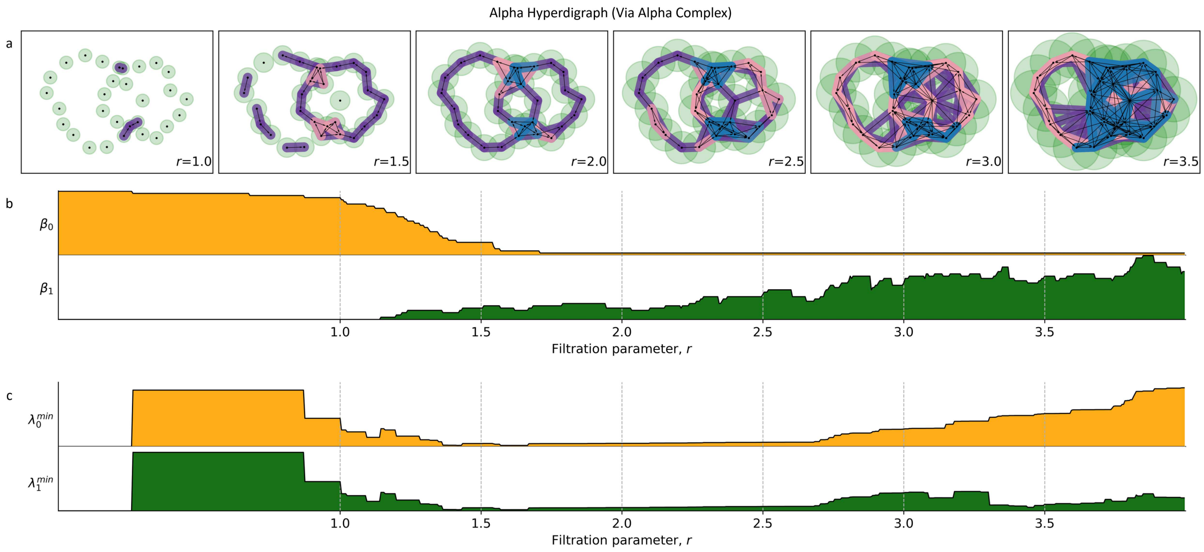

Figure 6: Illustration of the construction of alpha hyperdigraph via alpha complex with changing scale parameter for the point cloud in Figure 4**a**. **a** The constructed alpha hyperdigraphs for given scale parameter, i.e., $r = 1.0$, $r = 1.5$, $d = 2.0$, $d = 2.5$, $d = 3.0$, and $d = 3.5$. **c** The persistent Betti numbers of alpha hyperdigraphs, $\beta_i, i = 0, 1$. The verdical desh lines indicate the Betti numbers for given scale (filtration) parameters. **d** Representation of nonzero minimum non-harmonic spectra of the persistent topological hyperdigraph Laplacian at the 0th and 1st dimensions (λ_0^{min} and λ_1^{min}) for alpha hyperdigraph, highlighting their dependence on the filtration (scale) parameter d.

defined order of the points, the resulting topological hyperdigraph is constructed as a subset of the collection of these directed hyperedges. In this scenario, the VR hyperdigraph can be reduced to a hypergraph. Additionally, if we disregard the magnitude information of simplices, meaning all simplices are selected, then the VR hyperdigraph coincides with the VR complex. In this case, we denote the VR hyperdigraph by $\mathcal{VR}\vec{\mathcal{H}}_d(X)$.

The VR hyperdigraph $\mathcal{VR}\vec{\mathcal{H}}_d(X)$ captures the topological features of the underlying space at a scale determined by the parameter d. As d increases, as shown in Figure 5**a** and **b**, more simplices are added, resulting in the construction of more directed hyperedges. This provides information about the connectivity and holes in the space at different scales.

Similarly, the alpha hyperdigraph can be constructed on the alpha complex. For a metric space (M, d), let X be a finite point set in M. For a given parameter r, the alpha complex $\mathcal{A}_r$ is defined by

$$\mathcal{A}_r = \{S \subseteq X | \text{there is a disk of radius } r \text{ that covers } S\}. \tag{28}$$

Usually, people tend to regard the alpha complex as an abstract simplicial complex for computing its homology. However, the alpha complex itself possesses geometric structure. Considering functions $w : \mathcal{A}_r \to \mathbb{R}$ and $\varrho_n : (\mathcal{A}_r)_n \to S_{n+1}$, for any real number η, we can obtain the alpha hyperdigraph:

$$\mathcal{A}_r\vec{\mathcal{H}}_\eta = \{S \times \varrho_*(S) | w(S) \leq \eta, S \in \mathcal{A}_r\}. \tag{29}$$

Similar to the relationship between the alpha complex and the VR complex, alpha hyperdigraphs can capture distinct information compared to VR hyperdigraphs. If the maps $w : \mathcal{A}_r \to \mathbb{R}$ and $\varrho_n : (\mathcal{A}_r)_n \to S_{n+1}$ are chosen as trivial maps, the alpha hyperdigraph can also be reduced to the alpha complex.

In informal terms, the alpha complex involves the simplices that are close enough, meaning that one can find a disk of a given radius containing all the points in the simplex. The construction can also be derived from the 3-dimensional Voronoi diagram [63] or the Delaunay triangulation [64]. The alpha hyperdigraph is a collection of simplices in the alpha complex which are endowed with the corresponding orientation. If the orientation is chosen to follow a given order, the alpha hyperdigraph can be reduced to a hypergraph. Besides, if we choose all the simplices in the alpha complex as the collection, the alpha hyperdigraph can also be reduced to the alpha complex. In such case, the construction is denoted by $\mathcal{A}_r\vec{\mathcal{H}}(X)$.

For a given parameter $r > 0$, the alpha complex and alpha hyperdigraph, denoted as $\mathcal{A}_r(X)$ and $\mathcal{A}_r\vec{\mathcal{H}}(X)$, are constructed step by step as follows: 1. Include a vertex for each point in X. 2. For each subset S of X such that the maximum pairwise distance between points in S is less than or equal to r, include a simplex in the complex with vertices corresponding to the points in S. 3. Directed hyperedges are defined on all simplices using the predefined order of the set X, and the hyperdigraph $\mathcal{A}_r\vec{\mathcal{H}}(X)$ is then generated as the collection of these directed hyperedges.

The alpha hyperdigraph includes directed hyperedges for subsets of points that are in close proximity within the specified radius. As r increases, as shown in Figure 6**a**, more directed hyperedges are added to the hyperdigraph, capturing different levels of connectivity and features in the dataset. As illustrated in Figures 5**c**, **d** and 6**b**, **c**, the persistent attributes in higher dimensions of VR hyperdigraph Laplacians and alpha hyperdigraph Laplacians exhibit notable differences. However, for the 0-dimensional information, their persistent patterns remain the same.

Figures 5 and 6 illustrate the construction of the VR hyperdigraph and alpha hyperdigraph, respectively, with varying filtration parameters. Notably, both of these hyperdigraphs in this study are constructed based on the simplicial complex, incorporating the Vietoris-Rips (VR) complex and the alpha complex.

A.5 Supplementary tables

In the following section, we provide supplementary tables that offer additional data and insights pertinent to our study. Readers are encouraged to refer to these tables for a more detailed exploration of the topics covered in the main text.

In the finetuning stage of the Transformer model in TopoFormer-seq, Table 4 outlines three sets of hyperparameters, while keeping other settings constant at 10,000 training steps. According to the table, the optimal performance on the CASF-2007 dataset is achieved by TopoFormer$_s$-seq, with a batch size of 32 and a learning rate of 0.0008 during the finetuning stage. However, the performance remains nearly identical for the other two hyperparameter settings. For the CASF-2013 dataset, the best performance is observed with TopoFormer-seq, employing a batch size of 32 and a learning rate of 0.0008, resulting in a PCC of 0.816 and an RMSE of 1.367. Remarkably, even the least favorable hyperparameter setting, with a batch size of 32 and a learning rate of 0.001, yields a PCC of 0.815 and an RMSE of 1.373, a result very close to the optimal performance. For the CASF-2016 dataset, the superior performance is achieved by TopoFormer-seq with a batch size of 32 and a learning rate of 0.0008. All other hyperparameters result in the same PCC (0.864), albeit with slightly higher RMSE values of 1.160 and 1.157. The results indicate that the TopoFormer-seq and TopoFormer$_s$-seq models exhibit remarkable stability across different hyperparameter settings. To mitigate overfitting, a batch size of 32 and a learning rate of 0.0008 are consistently employed

Table 4: The PCCs and RMSEs of our TopoFormer-seq and TopoFormer$_s$-seq models on the three benchmarks of CASF-2007, CASF-2013, and CASF-2016 with different hyperparameter settings. The average of 400 experiments are reported in the table.

		TopoFormer-seq		TopoFormer$_s$-seq	
Hyperparameters	Datasets	PCC	RMSE	PCC	RMSE
Batch size 32 Learning rate 0.0008	CASF-2007	0.836	1.329	0.839	1.322
	CASF-2013	0.816	1.367	0.810	1.392
	CASF-2016	0.864	1.153	0.855	1.183
Batch size 64 Learning rate 0.0008	CASF-2007	0.837	1.333	0.837	1.335
	CASF-2013	0.816	1.373	0.812	1.387
	CASF-2016	0.864	1.160	0.858	1.184
Batch size 32 Learning rate 0.001	CASF-2007	0.837	1.331	0.838	1.329
	CASF-2013	0.815	1.373	0.810	1.390
	CASF-2016	0.864	1.157	0.856	1.183

for all scoring tasks in this study during validation and comparisons in the main text.

Table 5: The performance of recently proposed models is assessed through the evaluation of their PCCs(RMSEs) using various training datasets. To convert to the unit kcal/mol, a conversion factor of 1.3633 should be multiplied with the RMSEs in the table. Footnote ^{a} indicates variations in test dataset sizes, involving the PDBbind-v2013 core set (N = 180) and the PDBbind-v2016 core set (N = 276). Footnote ^{b} signifies the utilization of the PDBbind-v2016 core set (N = 290) as the testing dataset. We conducted performance testing on the CASF-2007 dataset using a training set comprising 18,904 protein-ligand complexes from the v2020 general set, excluding all core sets. The results show the best performance, with a Pearson correlation coefficient (PCC) of 0.853 and a root mean square error (RMSE) of 1.295.

Model	Training set	Core set (CASF-2013)	Core set (CASF-2016)
Ligand-based [38]	PDBbind-v2018(11663)	0.780^a	0.821^a
graphDelta [32]	PDBbind-v2018(8766)		0.87(1.05)^b
ECIF [33]	PDBbind-v2019(9299)		0.866(1.169)
OnionNet-2 [34]	PDBbind-v2019(>9000)	0.821(1.357)	0.864(1.164)
DeepAtom [35]	PDBbind-v2018(9383)		0.831(1.232)^b
SE-OnionNet [36]	PDBbind-v2018(11663)	0.812(1.692)	0.83
Deep Fusion [37]	PDBbind-v2016(9226)		0.803(1.327)^b
TopoFormer-Seq	PDBbind-v2020(18904)	0.832(1.301)	0.881(1.095)<br>0.883(1.086)^b

The performance of recently proposed models is presented in Table 5. Due to variations in the training sets utilized, direct comparisons among these models are not fair. The majority of these models employ a general set (or preprocessed general set) for training to enhance their performance on benchmarks. In this study, we introduced the TopoFormer-Seq model, trained on the PDBbind-v2020 general set, with exclusion of the core sets used for evaluation from the training process. As demonstrated in Table 5, the TopoFormer-Seq consistently exhibits the best performance across all benchmarks. For CASF-2007, the model achieves a PCC of 0.853 with an RMSE of 1.295, and for CASF-2013, the PCC is 0.832 with an RMSE of 1.301. Similarly, for CASF-2016, the TopoFormer-

Seq attains a PCC of 0.881 with a corresponding RMSE of 1.095. The model's performance on PDBbind-v2016 is also assessed, with a PCC of 0.883 and an RMSE of 1.086. It is important to note that, in this work, the performance of TopoFormer-Seq-2020 (trained on PDBbind-v2020 general set) is solely utilized to showcase the capability of the proposed model. The reported best performance in the main text adheres to the standard pipeline.

A.6 Supplementary figures

In this section, we present a series of supplementary figures that further elucidate and complement the findings discussed in the main text. Readers are encouraged to consult these figures for a richer understanding and visual representation of the concepts and results introduced in the primary manuscript.

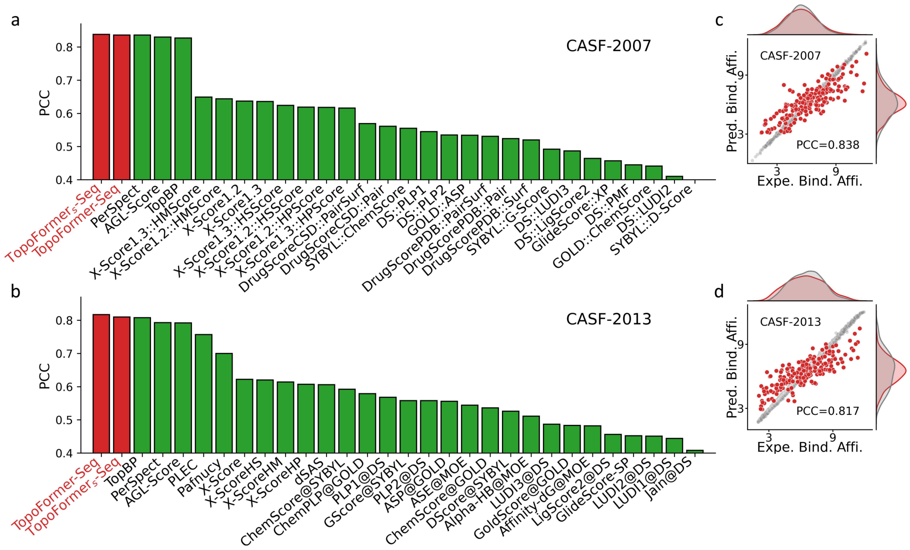

Figure 7: Performance of TopoFormer on scoring tasks for CASF-2007 and CASF-2013. **a**-**b** display the Pearson correlation coefficients (PCCs) of TopoFormers for protein-ligand complex binding affinity scoring. These are compared with models on the CASF-2007 (**a**), and CASF-2013 (**b**) benchmarks. The results from other methods are taking from refs ([25, 24, 26, 16, 27, 19, 28, 29, 30]), are in the green color. **c** to **d** showcase comparisons of predicted protein-ligand binding affinities and experimental results for the CASF-2007 (**c**, PCC=0.838), and CASF-2013 (**d**, PCC=0.817) benchmarks. Grey dots represent the training data, while red dots denote the test data.

Figure 10 presents the persistent attributes derived from the non-harmonic spectra of a persistent topological hypergraph at 0th and 1st dimensions. Specifically, the nonzero maximum spectra are denoted as $\lambda^{max}0$ and $\lambda^{max}1$ for the first and second dimensions, respectively. The persistent standard deviation of nonzero spectra is represented by λ_0^{std} and λ_1^{std} for the first two dimensions. Additionally, the values λ_0^{avg} and λ_1^{avg} correspond to the 0th and 1st dimensional persistent average values of nonzero spectra. The λ_0^{sum} and λ_1^{sum}, as shown in Figure 10**e**, indicate the persistent summation values of nonzero spectra of the persistent topological hyperdigraph Laplacian. Furthermore, we generated the persistent topological hypergraph homology for the Vietoris-Rips hypergraph, as depicted in Figure 10**f**. This figure displays persistent barcodes for homology groups H_0 and H_1 at the 0th and 1st dimensions, respectively. The topological invariants, specifically Betti numbers, correspond to the multiplicity of the zero eigenvalue of the topological hypergraph Laplacian for a given filtration parameter d. Notably, these persistent attributes vary with different filtration parameters, indicating the recording of both topological and geometric information in a multiscale manner.

The Figure 13 illustrates diverse topological representations for the C_α atoms in protein PDBID: 6L9D, with a cutoff distance set to 5 Å. In Figure 13**a**, the commonly used graph representation of the structure is depicted. Generalizing from the graph representation, the 0-simplices in

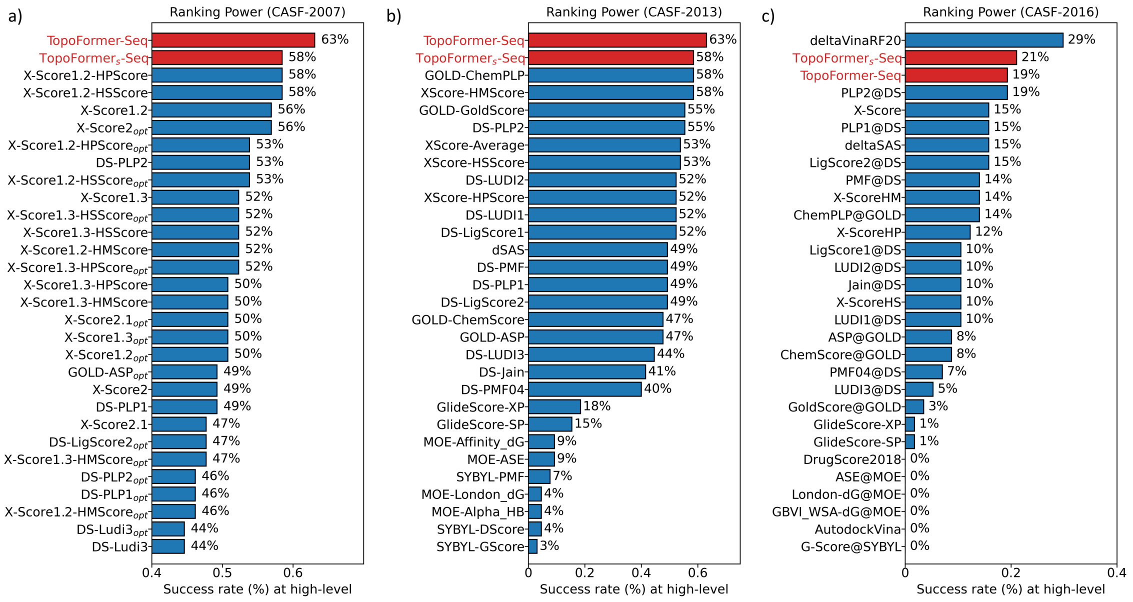

Figure 8: Performance of ranking power evaluated by the high-level success measurement compares with different scoring functions on CASF-2007, CASF-2013, and CASF-2016 benchmarks. The proposed TopoFormer-based models are plotted in the red color. The results of other methods, taken from refs ([25, 24, 26, 16, 27, 19, 30, 65]), are in the blue color

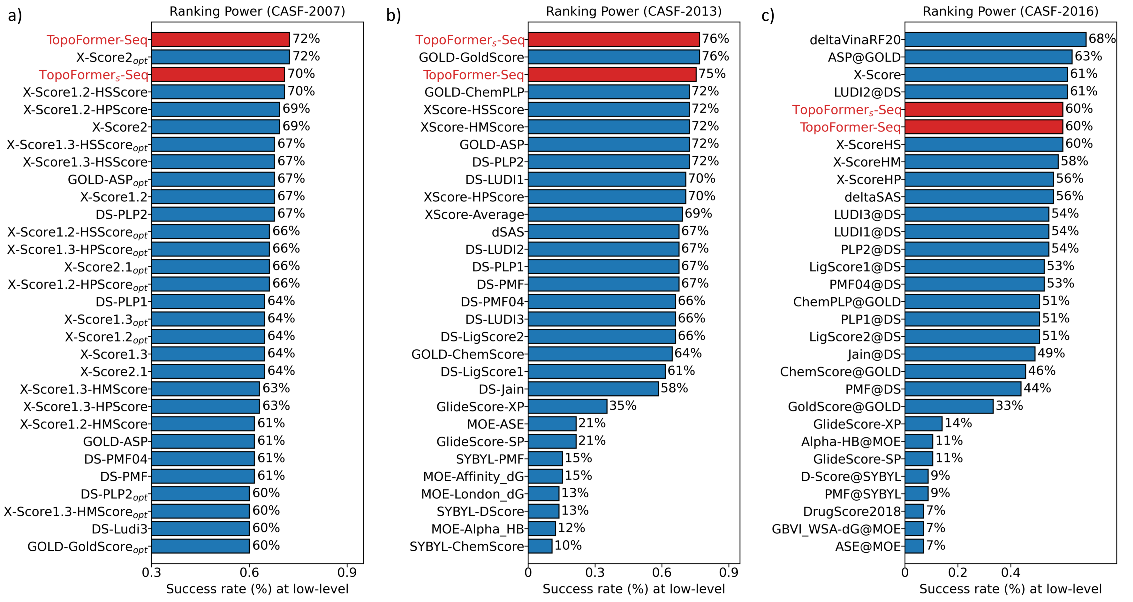

Figure 9: Performance of ranking power evaluated by the low-level success measurement compares with different scoring functions on CASF-2007, CASF-2013, and CASF-2016 benchmarks. The proposed TopoFormer-based models are plotted in the red color. The results of other methods, taken from refs ([25, 24, 26, 16, 27, 19, 30, 65]), are in the blue color.

the simplicial complex correspond to the vertices in the graph, while the 1-simplices represent edges with vertices from the graph, as shown in the second and third rows of Figure 13**a** and **b**. Additionally, higher-dimensional simplices in the simplicial complex provide more intricate information

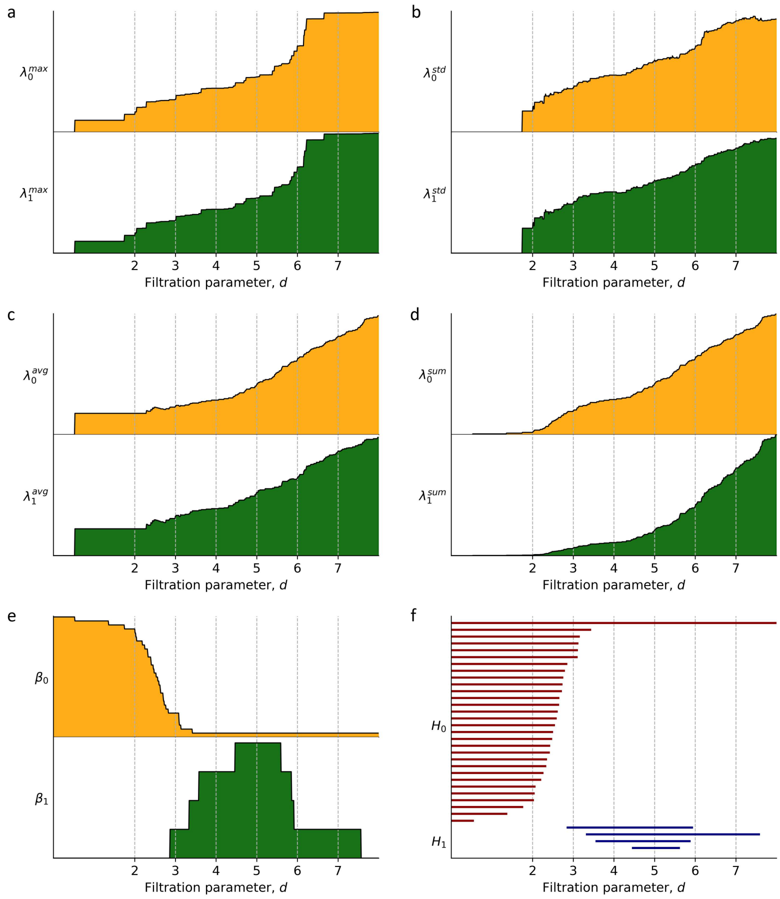

Figure 10: Comparison of persistent topological hyperdigraph Laplacian and persistent homology for the point cloud in Figure 4**a**. **a** Representation of nonzero maximum non-harmonic spectra of the persistent topological hyperdigraph Laplacian at the 0th and 1st dimensions (λ_0^{max} and λ_1^{max}), highlighting their dependence on the filtration (scale) parameter d. **b** Representation of standard deviation of nonzero spectra of the persistent topological hyperdigraph Laplacian at the 0th and 1st dimensions (λ_0^{std} and λ_1^{std}). **c** Representation of average value of nonzero spectra of the persistent topological hyperdigraph Laplacian at the 0th and 1st dimensions (λ_0^{avg} and λ_1^{avg}). **d** Representation of summation value of nonzero spectra of the persistent topological hyperdigraph Laplacian at the 0th and 1st dimensions (λ_0^{sum} and λ_1^{sum}). **e** Representation of multiplicity of zero of the persistent topological hyperdigraph Laplacian at the 0th and 1st dimensions (β_0 and β_1). **f** Visualization of barcodes for persistent homology groups H_0 and H_1 at the 0th and 1st dimensions, respectively, showcasing their variations with respect to the filtration parameter d.

about the structure; for instance, alpha helices can be roughly represented by 3-simplices.

Moving beyond simplicial complexes, hypergraphs offer a more generalized representation of the structure, as demonstrated in Figure 13**c**. Furthermore, with directional information, hyperdigraphs present an even more generalized view compared to simplicial complexes and hypergraphs. The hyperdigraph representation, along with different dimensional directed hyperedges, captures information at various levels, as illustrated in figure 13**d**. Notably, the representation via 3-directed hyperedges can also unveil the presence of an alpha helix.

To illustrate the power of proposed topological hyperdigraph and its Laplacian, two $B_7C_2H_9$ isomers with identical geometric structures, differing only in the positions of carbon atoms are used in the validation. Figures 14**a** and **b** show the molecular structures of these two $B_7C_2H_9$

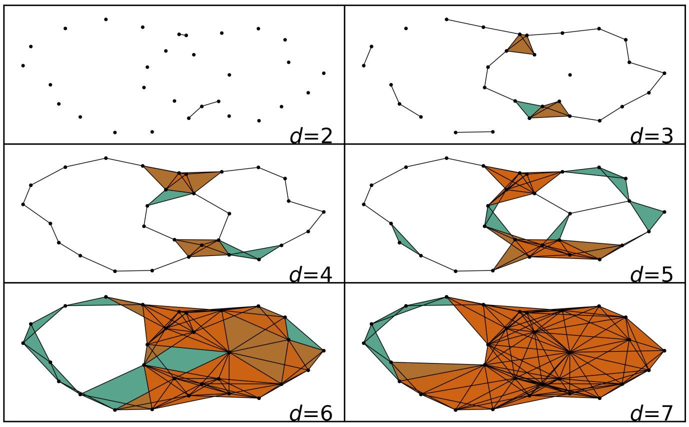

Figure 11: Illustration of how the changing filtration parameter leads to alterations in the connectivity of the point cloud in Figure 4**a**, resulting in the generation of a series of simplicial complex. The 2-simplices are triangles colored by the green. The 3-simplices are tetrahedrons colored by orange.

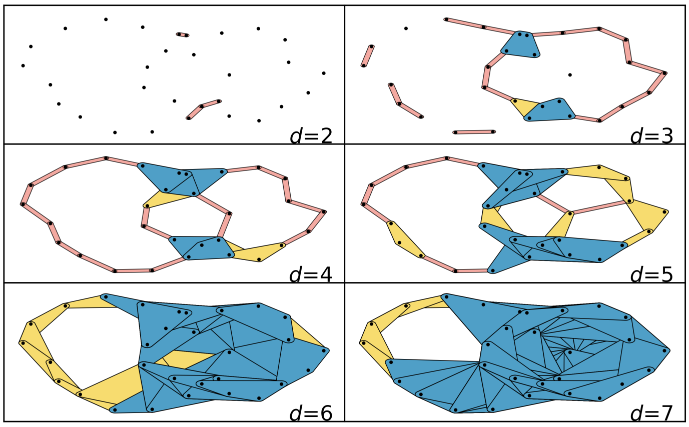

Figure 12: Illustration of how the changing filtration parameter leads to alterations in the connectivity of the point cloud Figure 4**a**, resulting in the generation of a series of hypergraph. The 1-hyperedge is represented by the light red area. The 2-hyperedge is represented by yellow area. The 3-hyperedge is represented by blue area.

isomers. Here, the structure without hydrogen atoms are considered in the analysis, as shown in Figure 14**c** and **d**. Figures 14**e**, **g**, and **i** are the simplicial complex, hypergraph, and hyperdigraph representations and their Laplacians' analysis results for structure 14**c**. Figures 14**f**, **h**, and **j** are the simplicial complex, hypergraph, and hyperdigraph representations and their Laplacians' analysis results for other structure in Figure 14**d**. While only carbon atoms are changed in the structures 14**c** and **d**, the Laplacians analysis for simplicial complex and hypergraph can not classify these two structures. For hyperdigraph, because the directed hyperedge can be used to encode the non-symmetry and non-balance relations, the changing position of carbon atoms can be captured by the directed hyperedge, which result different topological hyperdigraph Laplacians. So that either the multiplicity of zero eigenvalue of these Laplacians (β_0, β_1, and β_2) or the minimum nonzero

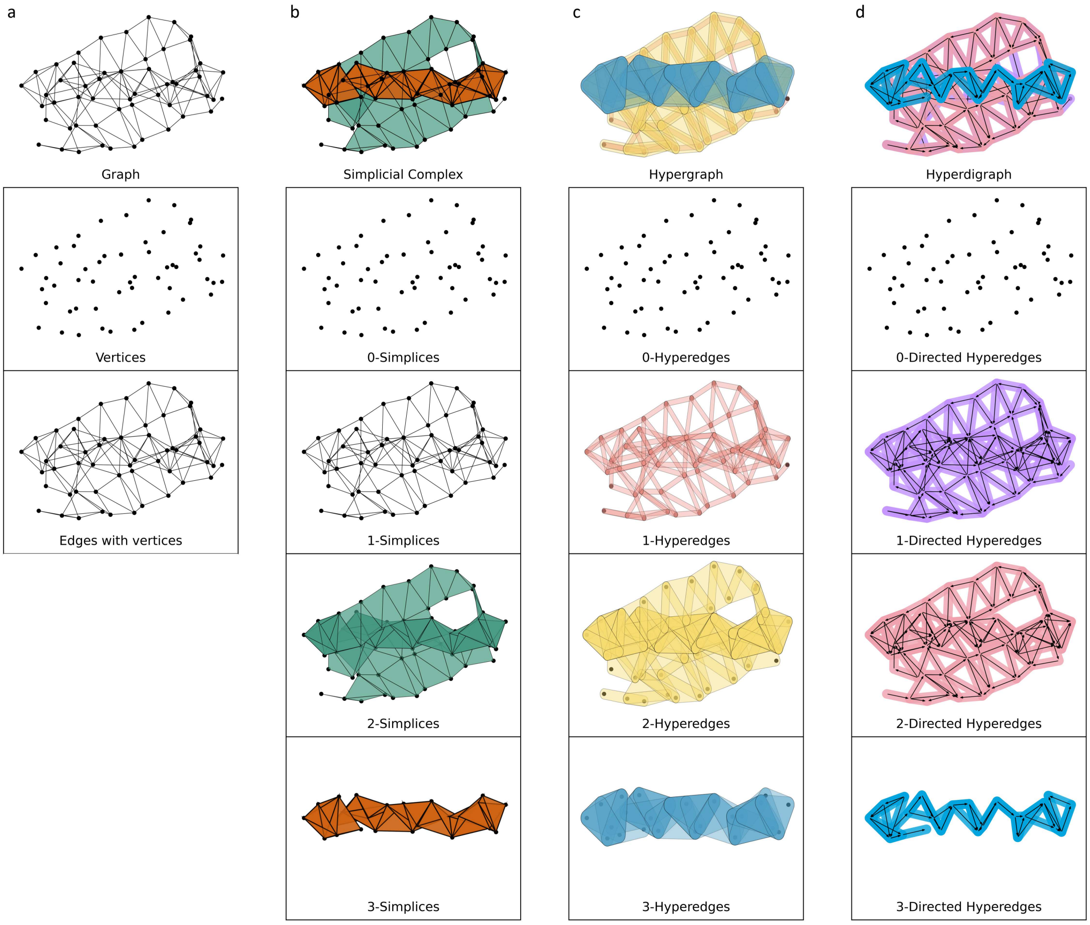

Figure 13: Illustration of Different Representations for C_α Atoms in Protein PDBID: 6L9D. **a** The graph representation of the structure. **b** The simplicial complex representation of the structure, including a list of 0-simplices, 1-simplices, 2-simplices, and 3-simplices in the complex. **c** The hypergraph representation of the structure, along with a list of 0, 1, 2, and 3-hyperedges within the hypergraph. **d** The hyperdigraph representation of the structure, providing a listing of 0, 1, 2, and 3-directed hyperedges in the hyperdigraph.

spectra of there Laplacians (λ_0^{min}, λ_1^{min}, and λ_2^{min}) can distinct these two structures.

To assess the efficacy of the proposed topological hyperdigraph and its Laplacian, we employ two $B_7C_2H_9$ isomers sharing identical geometric structures but differing solely in the positions of carbon atoms. Figures 14**a** and **b** illustrate the molecular structures of these isomers. In the analysis, we consider the structures without hydrogen atoms, as depicted in Figures 14**c** and **d**. Figures 14**e**, **g**, and **i** present the analysis results for the simplicial complex, hypergraph, and hyperdigraph representations, along with their Laplacians, for the structure in Figure 14**c**. Similarly, Figures 14**f**, **h**, and **j** display the analysis results for the other structure in Figure 14**d**. Despite the alteration being limited to the carbon atoms in structures shown in Figures 14**c** and **d**, the Laplacian analysis of simplicial complex and hypergraph representations fails to differentiate these two structures. In contrast, the hyperdigraph, leveraging directed hyperedges to encode non-symmetry and non-balance relations, effectively captures the changing positions of carbon atoms. This results in distinct topological hyperdigraph Laplacians, reflected in either the multiplicity of zero eigenvalues (β_0, β_1, and β_2) or the minimum nonzero spectra of these Laplacians (λ_0^{min}, λ_1^{min},

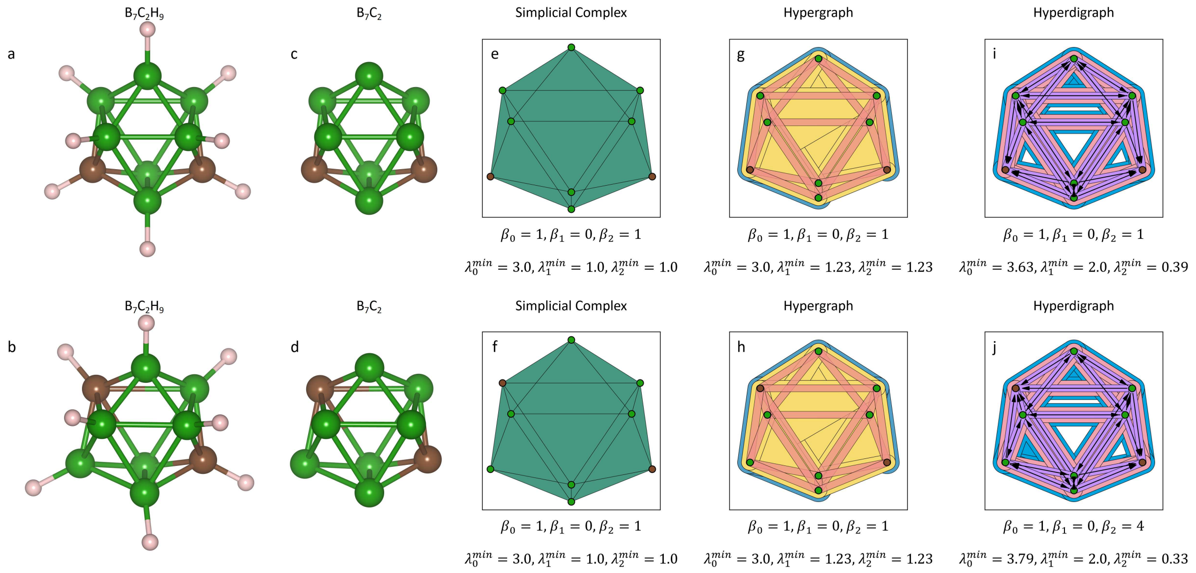

Figure 14: Illustration of the different Laplacian analysis for two $B_7C_2H_9$ isomers. **a** and **b** Two $B_7C_2H_9$ isomers with identical geometric structures, differing only in the positions of carbon atoms. **c** and **d** The structural representations of $B_7C_2H_9$ after the removal of hydrogen atoms. **e** and **f** The simplicial complex representations of structures **c** and **d**. Carbon atoms are highlighted in coffee color, while boron atoms are shown in light green. The 2-simplices are shaded in green. Notably, the corresponding topological invariants and non-harmonic spectra are consistent, with $\beta_0 = 1$, $\beta_1 = 0$, and $\beta_2 = 1$, as well as $\lambda_0^{min} = 3.0$, $\lambda_1^{min} = 1.0$, and $\lambda_2^{min} = 1.0$. **g** and **h** The hypergraph representations of structures **c** and **d**. The 1-hyperedges are colored in light red, and 2-hyperedges are colored yellow, while 3-hyperedges are shaded in blue. The corresponding topological invariants and non-harmonic spectra are consistent, with $\beta_0 = 1$, $\beta_1 = 0$, and $\beta_2 = 1$, as well as $\lambda_0^{min} = 3.0$, $\lambda_1^{min} = 1.23$, and $\lambda_2^{min} = 1.23$. **i** The hyperdigraph representation of structure **c**. The 1-directed hyperedges are colored in purple, the 2-directed hyperedges are pink, and the 3-directed hyperedges are blue. The corresponding topological invariants are $\beta_0 = 1$, $\beta_1 = 0$, and $\beta_2 = 1$. The minimum non-zero non-harmonic spectra are $\lambda_0^{min} = 3.63$, $\lambda_1^{min} = 2.0$, and $\lambda_2^{min} = 0.39$. **j** The hyperdigraph representation of structure **c**. The 1-directed hyperedges are colored in purple, the 2-directed hyperedges are pink, and the 3-directed hyperedges are blue. The corresponding topological invariants are $\beta_0 = 1$, $\beta_1 = 0$, and $\beta_2 = 4$. The minimum non-zero non-harmonic spectra are $\lambda_0^{min} = 3.79$, $\lambda_1^{min} = 2.0$, and $\lambda_2^{min} = 0.33$. The difference in Charts **i** and **j** demonstrates that topological hyperdigraph Laplacians can distinguish these two isomers.

and λ_2^{min}). Consequently, these topological features successfully distinguish two structures.
